# Differential accumulation of pelargonidin glycosides in petals at three different developmental stages of the orange-flowered gentian (*Gentiana lutea* L. var. *aurantiaca*)

**DOI:** 10.1371/journal.pone.0212062

**Published:** 2019-02-11

**Authors:** Gianfranco Diretto, Xin Jin, Teresa Capell, Changfu Zhu, Lourdes Gomez-Gomez

**Affiliations:** 1 Italian National Agency for New Technologies, Energy and Sustainable Development, Casaccia Research Centre, Rome, Italy; 2 Departament de Producció Vegetal i Ciència Forestal, Universitat de Lleida-Agrotecnio Center, Lleida, Spain; 3 School of Life Sciences, Changchun Normal University, Changchun, China; 4 Instituto Botánico, Departamento de Ciencia y Tecnología Agroforestal y Genética, Facultad de Farmacia, Universidad de Castilla-La Mancha, Campus Universitario, Albacete, Spain; Zhejiang University, CHINA

## Abstract

Corolla color in *Gentiana lutea* L. exhibits a yellow/orange variation. We previously demonstrated that the orange petal color of *G*. *lutea* L. var. *aurantiaca* is predominantly caused by newly synthesized pelargonidin glycosides that confer a reddish hue to the yellow background color, derived from the carotenoids. However, the anthocyanin molecules of these pelargonidin glycosides are not yet fully identified and characterized. Here, we investigated the regulation, content and type of anthocyanins determining the petal coloration of the orange-flowered *G*. *lutea* L. var. *aurantiaca*. Anthocyanins from the petals of *G*. *lutea* L. var. *aurantiaca* were characterized and quantified by HPLC-ESI-MS/MS (High-performance liquid chromatography-electrospray ionization-tandem mass spectrometry) coupled with a diode array detector in flowers at three different stages of development (S1, S3 and S5). Eleven pelargonidin derivatives were identified in the petals of *G*. *lutea* L. var. *aurantiaca* for the first time, but quantitative and qualitative differences were observed at each developmental stage. The highest levels of these pelargonidin derivatives were reached at the fully open flower stage (S5) where all anthocyanins were detected. In contrast, not all the anthocyanins were detected at the budlet stage (S1) and mature bud stage (S3) and those corresponded to more complex pelargonidin derivatives. The major pelargonidin derivatives found at all the stages were pelargonidin 3-*O*-glucoside, pelargonidin 3,5-*O*-diglucoside and pelargonidin 3-*O*-rutinoside. Furthermore, the expression of *DFR* (*dihydroflavonol 4-reductase*), *ANS* (*anthocyanidin synthase*), *3GT* (*UDP-glucose*:*flavonoid 3-O-glucosyltransferase*), *5GT* (*UDP-glucose*:*flavonoid 5-O-glucosyltransferase*) and *5AT* (*anthocyanin 5-aromatic acyltransferase*) genes was analyzed in the petals of three developmental stages, showing that the expression level of *DFR*, *ANS* and *3GT* parallels the accumulation of the pelargonidin glucosides. Overall, this study enhances the knowledge of the biochemical basis of flower coloration in *Gentiana* species, and lays a foundation for breeding of flower color and genetic variation studies on *Gentiana* varieties.

## Introduction

Polymorphism in flower color has been associated with the preferences of pollinators for certain colorations, primarily due to the ability of pollinators to perceive and distinguish among different hues [1–3]. The final color of a flower is determined by the presence of different pigments, mainly carotenoids, flavonoids or betalains [4].

**Fig 1:**
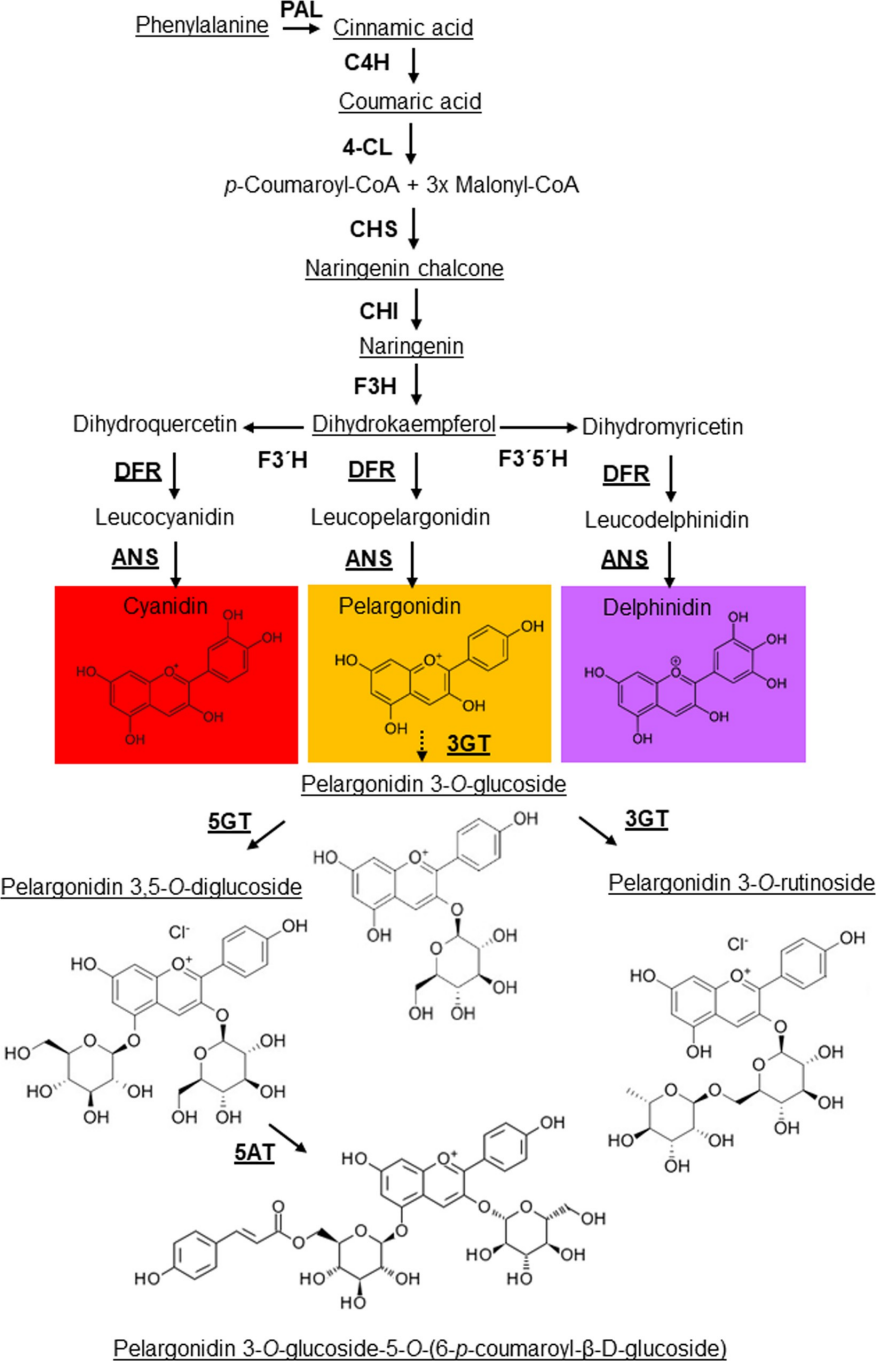
The gentian phenylpropanoid pathway. Anthocyanidins (pelargonidin, cyanidin, and delphinidin) are modified by the addition of sugars and other moieties to form anthocyanins in a species-specific manner. Pink-flowered gentians contain gentiocyanin in their petals, and blue-flowered gentians mainly gentiodelphin, whereas orange-flowered gentian accumulates exclusively pelargonidin glycosides. Abbreviations: ANS, anthocyanidin synthase; 5AT, anthocyanin 5-aromatic acyltransferase; CHI, chalcone isomerase; C4H, cinnamic acid 4-hydroxylase; 4CL, 4-coumarate:CoA ligase; CHS, chalcone synthase; DFR, dihydroflavonol 4-reductase; DHK, Dihydrokaempferol; DHM, Dihydromyricetin; DHQ, Dihydroquercetin; F3H, flavanone 3-hydroxylase; F3'H, flavonoid 3'-hydroxlase; F3'5'H, flavonoid 3',5'-hydroxlase; 3GT, UDP-glucose:flavonoid 3-*O*-glucosyltransferase; 5GT, UDP-glucose:flavonoid 5-*O*-glucosyltransferase; PAL, phenylalanine ammonia-lyase. Underlined names indicate enzymes/metabolites whose encoding gene expression/accumulation has been investigated in the present study.

Anthocyanins are water-soluble pigments derived from the flavonoid branch of the phenylpropanoid pathway [5–7]. The genes encoding for enzymes of the anthocyanin pathway are grouped in two classes (Fig 1): early biosynthetic genes, which are common to other flavonoids, encoding enzymes including chalcone synthase (CHS) which is responsible for the formation of naringenin chalcone from 4-coumaroyl CoA and malonyl CoA substrates, chalcone isomerase (CHI) which generates flavones, flavanone 3-hydroxylase (F3H) which produces dihydrokaempferol, flavonoid 3′-hydroxylase (F3′H) and flavonoid 3′,5′-hydroxylase (F3′5′H) which generate dihydroquercetin and dihydromyricetin respectively; and late biosynthetic genes, specific to the anthocyanin pathway, encoding enzymes including dihydroflavonol 4-reductase (DFR) and anthocyanidin synthase (ANS) [5–7]. Differences in the hydroxylation of the B-ring of the anthocyanin skeleton confer specific variants in the color range of anthocyanidins: pelargonidins, being orange to red; cyanidins, red to red-purple (by the activity of F3′H); and delphinidins, being red-purple to blue (by the activity of F3′5′H), depending on many other factors [4,7,8]. The resulting anthocyanidin structures are inherently unstable. Therefore, these pigments accumulate exclusively as glycosylated forms, where C_3_ is linked through oxygen to a sugar residue, most frequently glucose. UDP-glucose:flavonoid 3-*O*-glucosyltransferase (3GT) catalyzes the 3-*O*-glucosylation of anthocyanidins as well as other flavonoids [4]. The generated glycosylated anthocyanins at the 3-position serve as substrates for further modifications such as glycosylation at the 5-position (Fig 1), by the action of UDP-glucose:flavonoid 5-*O*-glucosyltransferase (5GT) [9,10]. The glycosylation reactions provide sugar residues for decoration with acyl groups by the activity of anthocyanin acyltransferase, which can incorporate aromatic acids such as *p*-coumaric, caffeic, ferulic, sinapic, gallic or *p*-hydroxybenzoic acids, and/or aliphatic acids including malonic, malic, acetic, succinic or oxalic acids. These acyl substituents are commonly bound to the C_3_ sugar, esterified to the 6-OH or much less frequently to the 4-OH group of the sugars [11]. Other modification also occurs, such as acylation of the glucose moiety at the 5*-*position of anthocyanin catalyzed by anthocyanin 5-aromatic acyltransferase (5AT) [12]. Both glycosyltransferases and acyltransferases contribute to the extensive range of natural anthocyanins identified to date, which differ in their side chain decorations, affecting color and increasing pigment stability [6].

The genus *Gentiana* includes over 400 species [13], with a huge range of flower colouration including orange, pink, red, magenta, purple, and blue to blue-black. This color variation is mainly due to the accumulation of anthocyanins and/or carotenoids. Most gentian species of commercial interest in the flower market include those with an intense blue coloration, due to the accumulation of major blue gentiodelphin, a polyacylated delphinidin-type anthocyanin, and minor red gentiocyanin, a cyanidin-derived anthocyanin in petals of *G*. *triflora* [14,15] and those with pink coloration, associated to the exclusive accumulation of red gentiocyanins in petals of *G*. *scabra* [16,17]. Both *G*. *triflora* and *G*. *scabra* do not accumulate carotenoids in the petals [18]. *G*. *leucomelaena* manifests dramatic flower color polymorphism, with both blue- and white-flowered individuals both within a population and on an individual plant [19]. Flower color of *G*. *lutea* varies among populations, ranging from yellow, orange to almost red shades [20]. The yellow petals of *G*. *lutea* L. var. *lutea* are due to the exclusive accumulation of abundant amounts of β-carotene and xanthophylls, while the orange color of the flowers of *G*. *lutea* L. var. *aurantiaca* (M. Laínz) is caused by newly synthesized pelargonidin glycosides that confer a reddish hue to the yellow background color, derived from the carotenoids [21]. However, the anthocyanin molecules of these pelargonidin glycosides in petals of orange-flowered *G*. *lutea* L. var. *aurantiaca* are not yet fully identified and characterized.

To date, the most used methods for separation and quantitation of anthocyanins are high-performance liquid chromatography (HPLC) with UV-Vis or diode array detectors (DAD) [3,22–24]. However, reverse-phase HPLC coupled to mass spectrometry (MS) offers the most powerful approach for identification of individual anthocyanins [25,26]. In this study, we identified and characterized the pelargonidin derivatives present in the petals of *G*. *lutea* L. var. *aurantiaca*, and their evolution during the development of the petals by using HPLC-DAD in tandem with electrospray ionization-tandem mass spectrometry (ESI-MS/MS). We also isolated the key late anthocyanin biosynthesis gene (*5GT* and *5AT*) fragments (cDNAs), and analyzed the expression levels of *DFR*, *ANS*, *3GT*, *5GT* and *5AT* to determine the molecular mechanism responsible for the biosynthesis and accumulation of the pelargonidin glycosides in petal of *G*. *lutea* L. var. *aurantiaca* during flower development.

## Materials and methods

### Chemicals

All the chemicals and solvents were liquid chromatography–mass spectrometry (LC/MS) grade (LiChrosolv, Merck). Water was reverse osmosis Milli-Q water (18.2 MΩ) (Millipore, USA). The standard pelargonidin was purchased from Sigma (St. Louis, MO, USA). Pelargonidin 3-*O*-glucoside, pelargonidin 3,5-*O*-diglucoside, naringenin, cinnamic acid, coumaric acid, and dihydrokaempferol were purchased from TransMIT (Marburg, Germany). Formononetin was purchased from Indofine Chemicals (indofinechemical.com).

### Plant materials

*Gentiana lutea* L. var. *aurantiaca* flowers were collected from Torrestío, León (Spain) in the Eurosiberian phytogeographic region (43º 03' N; 6º 04' W; 1600 m above sea level). All samples were collected in August of 2013. After harvest, all samples were frozen in liquid nitrogen and stored at -80°C until use. Petal tissues were collected at three stages of floral development, freeze-dried and stored at -80°C prior to pigment analysis. Each developmental stage was defined in terms of flower bud length as previously described [21]: stage 1 (S1), budlet stage, petals <1.5 cm long with hard bud; stage 3 (S3), mature bud stage, petals 2.5–3.5 cm long with petals slightly loosened; and stage 5 (S5), fully open flower stage.

### Anthocyanin and precursor extraction and identification

All the extractions were performed in triplicate. Freeze-dried petals were ground into a powder and homogeneously ground using 500 μL of 85:15 methanol:1 N HCl, and left in the dark at 4°C for 24 h. The extracted liquid was filtered with 0.22 μm membrane filter and used for HPLC-DAD analyses as described previously [21].

Anthocyanins were extracted from 10 mg of homogeneously ground petal tissue, using 500 μL of 85:15 methanol:1 N HCl with 2 μg L^-1^ of formononetin (Sigma-Aldrich, USA) as internal standard for quantitative analysis. After shaking for 12 hours and centrifugation for 10 minutes at 15,300 *g* at 25°C, 0.25 mL of supernatants were transferred to new Eppendorf vials and dried. Samples were then resuspended in 0.1 mL 75% methanol (plus 0.1% formic acid) and centrifuged (10 minutes at 15,300 *g* at 25°C). Finally, 70 μL of supernatants were transferred to HPLC vials for MS (mass spectrometry) analysis, and 5 μL of extracts were injected to the HPLC-DAD/MS. HPLC analysis was performed using a C18 Luna column (Phenomenex, Aschaffenburg, Germany) (150 × 2.0 mm; 3 μm). 10 μL of each extract were injected at a flow of 0.2 mL min^−1^. The mobile phases were: 0.1% formic acid (A) in water and acetonitrile plus 0.1% formic acid (B). The method used for separation was: 95% A: 5% B for 1 min, and a linear gradient to 25% A: 75% B over 40 min, plus an additional 2 min, before returning to the initial conditions for 18 min. Detection was performed continuously from 200 to 600 nm with an online Accela Surveyor photodiode array detector (PDA; Thermo Fisher Scientific). Mass spectrometry analysis was performed using a quadrupole-Orbitrap Q-exactive system (ThermoFisher scientific, USA), as previously described [27] with slight modifications. Metabolite ionization was carried out with a heated electrospray ionization (HESI) source operating in positive ion mode, and nitrogen was used as sheath and auxiliary gas (45 and 15 units, respectively). Mass spectrometer parameters were as follows: capillary and vaporizer temperatures 30°C and 270°C, respectively, discharge current 4.0 KV, probe heater temperature at 370°C, S-lens RF level at 50 V. The acquisition was carried out in the 110/1600 m/z scan range, with the following parameters: resolution 70,000, microscan 1, AGC target 1e6, and maximum injection time 50. A first full scan MS with data-dependent MS/MS fragmentation was used to identify the anthocyanins in the *G*. *lutea* L. var *aurantiaca* flower extracts. Subsequently, a single ion monitoring (SIM) with targeted MS/MS fragmentation was used to identify anthocyanins for which Data-dependent MS/MS fragmentation was not successful, and to further validate the tentative identifications. Data were analyzed using the Xcalibur 3.1 software (ThermoFisher scientific, USA). Metabolites were identified as M+ adducts, based on their accurate masses (m/z) and MS fragmentation, using both in house database and public sources (e.g. KEGG, MetaCyc, ChemSpider, PubChem, Metlin, Phenol-Explorer). Relative abundances of the metabolites studied were calculated using the Xcalibur 3.1 software (ThermoFisher scientific, USA). The two most abundant fragments per compound were used to determine the relative abundances, and data were normalized through dividing each peak area by the value of the internal standard peak area [28]. Data are presented as means and standard deviation of at least three independent biological replicates.

For precursor analyses 15 mg of freeze-dried, homogenized *G*. *lutea* L. *var*. *aurantiaca* petals were extracted with 0.75 mL cold 75% (v/v) methanol, with 0.1% (v/v) formic acid and spiked with 5 μg mL^-1^ formononetin. Samples were shaken for 40 min at 20 Hz using a Mixer Mill 300 (Qiagen), before centrifugation for 15 min at 15,300 *g*. Finally, 0.6 mL of supernatants was placed in the HPLC tubes. HPLC-DAD/MS analyses were carried out using a Q-exactive quadrupole Orbitrap mass spectrometer (ThermoFisher Scientific), operating in positive/negative heated electrospray ionization (HESI), and coupled to an Ultimate HPLC-DAD system (Thermo Fisher Scientific, Waltham, MA). HPLC conditions were as in the section 4.4.ESI-MS ionization was carried out as follows: sheath and aux gas flow rate set at 40 and 25 units, respectively; vaporizer and capillary temperature were used at 250 and 30°C, discharge current was set at 4.5 μA and S-lens RF level at 50. Acquisition was achieved as previously reported. Metabolite identification was carried out using a single ion monitoring (SIM) scan mode, with targeted MS/MS fragmentation, and by comparing chromatographic and spectral properties with authentic standards. Data were analyzed using Xcalibur 3.1 software (ThermoFisher scientific, USA). Metabolites were quantified in a relative way by normalization on the internal standard amounts. Data are presented as means and standard deviation of at least 3 independent biological replicates.

### Cloning of UDP-glucose:Flavonoid 5-*O*-glucosyltransferase (5GT) and anthocyanin 5-aromatic acyltransferase (5AT) gene fragments from petals of *G*. *lutea* L. *var*. *aurantiaca*

Three grams of leaf, petals at S1, S3 and S5 stages, respectively, or mixed petals from S1, S3 and S5 stages (1 g from each stage) were ground into fine powder using mortar and pestle with liquid nitrogen. From theses samples, 100 mg samples were used to isolate total RNA for each extraction. Total RNA was isolated using the RNeasy Plant Mini Kit (Qiagen, Valencia, California, USA) and DNA was removed with DNase I (RNase-free DNase Set, Qiagen). The integrity of RNA was confirmed by gel electrophoresis in a denaturing 1.2% (w/v) agarose gel containing formaldehyde. Total RNA was quantified using a Nanodrop 1000 spectrophotometer (Thermo Scientific, Vernon Hills, Illinois, USA), and 1 μg total RNA was used as template for first strand cDNA synthesis with QuantiTect Reverse Transcription Kit (Qiagen) in a 20 μL total reaction volume, following the manufacturer’s recommendations.

A 2 μL aliquot of the first strand cDNA product with isolated total RNAs from mixed petals of S1, S3 and S5 stages of *G*. *lutea* L. *var*. *aurantiaca* was used in a 50 μL RT-PCR reaction containing 1x Green GoTaq Reaction Buffer (Promega, Madison, WI, USA), 0.2 mM of each dNTP, 0.5 μM of each primer, and 1.25 U GoTaq DNA Polymerase (Promega, Madison, WI, USA). Reverse transcription polymerase chain reaction (RT-PCR) was performed using primer sequences designed on *5GT* and *5AT* sequences from *G*. *triflora*, to amplify partial protein coding sequences of cDNAs for *5GT* and *5AT* from *G*. *lutea* L. var. *aurantiaca*. The primer sequences for the *5GT* were 5´-CTGCTTCCTTGGGCTGCCGATGTGGCT-3´ (forward primer) and 5´-TGCGGATGGAATTCAACGCTGGAGAGC-3´ (reverse primer). The primer sequences for the *5AT* were 5´-CCGCTCGTAGCCGTGCAAGTAACCGTTTTTCCT-3´ (forward primer) and 5´-GATTTTGGATGGGGAAAGCCTGCAAAATTTGA-3´ (reverse primer). The PCR conditions for *5GT* and *5AT* genes involved heating the reaction to 95°C for 3 min, followed by 35 cycles at 95°C for 45 s, 60°C for 45 s and 72°C for 90 s, and a final extension step at 72°C for 10 min. The resulting expected size products were purified from a 1.0% w/v agarose gel using the Geneclean II Kit (BIO1 101 Systems, Solon, OH, USA) and cloned in pGEM-T easy vector (Promega, Madison, WI, USA) for sequencing with the Big Dye Terminator v3.1 Cycle Sequencing Kit on a 3130x1 Genetic Analyzer (Applied Biosystems, Foster City, CA, USA). Four and six independent clones provided the identical sequences for isolated *5GT* and *5AT* gene fragments, respectively, from 20 and 26 independent sequenced clones. The nucleotide sequences were translated to the respective deduced amino acid sequences using Vector NTI software, which was also used for alignments of cDNA and deduced amino acid sequences, respectively. Both cloned *5GT* and *5AT* showed sequence similarity to previously characterized anthocyanin structural genes and BLAST searches against the National Center for Biotechnology Information (NCBI) nucleotide and protein databases were used to confirm homology with previously characterized *5GT* and *5AT* genes from higher plants [12].

### qRT-PCR analysis of *DFR*, *ANS*, *3GT*, *5GT* and *5AT* expression in leaf, S1, S3 and S5 petals of *G*. *lutea* L. var. *aurantiaca*

From the above *5GT* and *5AT* partial cDNA sequences, gene-specific primers were designed for these two genes, in order to perform expression analysis by quantitative real-time RT-PCR (qRT-PCR). The primers for the *5GT* were 5´-GCCTTGCAAGCAATCCCAAA-3´ (forward primer) and 5´-CAACCATCCATGGCAGTCCT-3´ (reverse primer). The primers for the *5AT* were 5´-AGCTGTTGGGGATGCCATTG-3´ (forward primer) and 5´-CGATCCACTAATCCCGAGCAA-3´ (reverse primer). Gene-specific oligonucleotides for *DFR*, *ANS*, *3GT* and *UBQ* (ubiquitin gene) were the same as reported previously [21]. Specific primers that amplified a single fragment of no more than 200 bp were designed in regions of the coding sequences isolated from orange-flowered *G*. *lutea* L. var *aurantiaca* petals. qRT-PCRs were carried out using a BioRad CFX96 system. Each 25 μL reaction volume comprised 5 ng of cDNA, 1x iQ SYBR green supermix (BioRad, Hercules, CA, USA), and 5 μM of forward and reverse primers. Relative expression levels were calculated on the basis of serial dilutions of cDNA (100–0.16 ng), which were used to generate standard curves for each gene. Triplicate amplifications (biological replicates) were performed in 96-well optical reaction plates by first heating to 95°C for 5 min, followed by 40 cycles at 95°C for 30 s, 58°C for 30 s and 72°C for 30 s. Amplification specificity was confirmed by product melt curve analysis over the temperature range 50–90°C with fluorescence acquired after every 0.5°C increase. The fluorescence threshold value and gene expression data were calculated with BioRad CFX96 software. Data were calculated from three biological replicates with at least three technical replicates for each biological replicate and with error bars representing the standard deviation. Amplification efficiencies were compared by plotting the ΔCt values of different primer combinations of serial dilutions against the log of starting template concentrations using the CFX96 software. The Ct values were adjusted to the standard curves and normalized against the levels of ubiquitin (*UBQ*) housekeeping gene [21].

### Bioinformatics analyses

Correlation analyses of transcript and metabolite data were performed as previously described [29,30]. Briefly, Pearson correlation coefficients for each data pair (gene-gene, metabolite-metabolite, gene-metabolite) were compiled in Excel and subjected to significance analysis by SPSS software; only correlations showing correlations with P values ≤0.05 were further processed. Subsequently, correlation matrix was generated using R version 2.10.0, and colored with the Morpheus software (https://software.broadinstitute.org/morpheus/). Correlation network analysis was performed using R to generate a network correlation input file, which was finally imported in Cytoscape version 2.6.2 (http://www.cytoscape.org/), and visualize using a Prefuse force-directed layout. Node strength (ns) and network strength (NS) were calculated as reported previously [31,32], and represent the average of all the ρs (Pearson correlation coefficients) yielded by each node, and the average of all the node strengths in the network, respectively.

## Results and discussion

### Identification of anthocyanins in petals of three developmental stages of *G*. *lutea* L. var. *aurantiaca*

HPLC reverse phase C18 columns have been extensively used for the separation of anthocyanins in different plant species [33–35]. The acidic methanol extracts of the petals of *G*. *lutea* L. var. *aurantiaca* showed mixtures of several peaks (S1 Fig). The anthocyanins corresponding to the peaks (500 nm) observed in the HPLC-DAD chromatogram were characterized and quantified by HPLC-ESI-MS/MS analyses.

**Fig 2:**
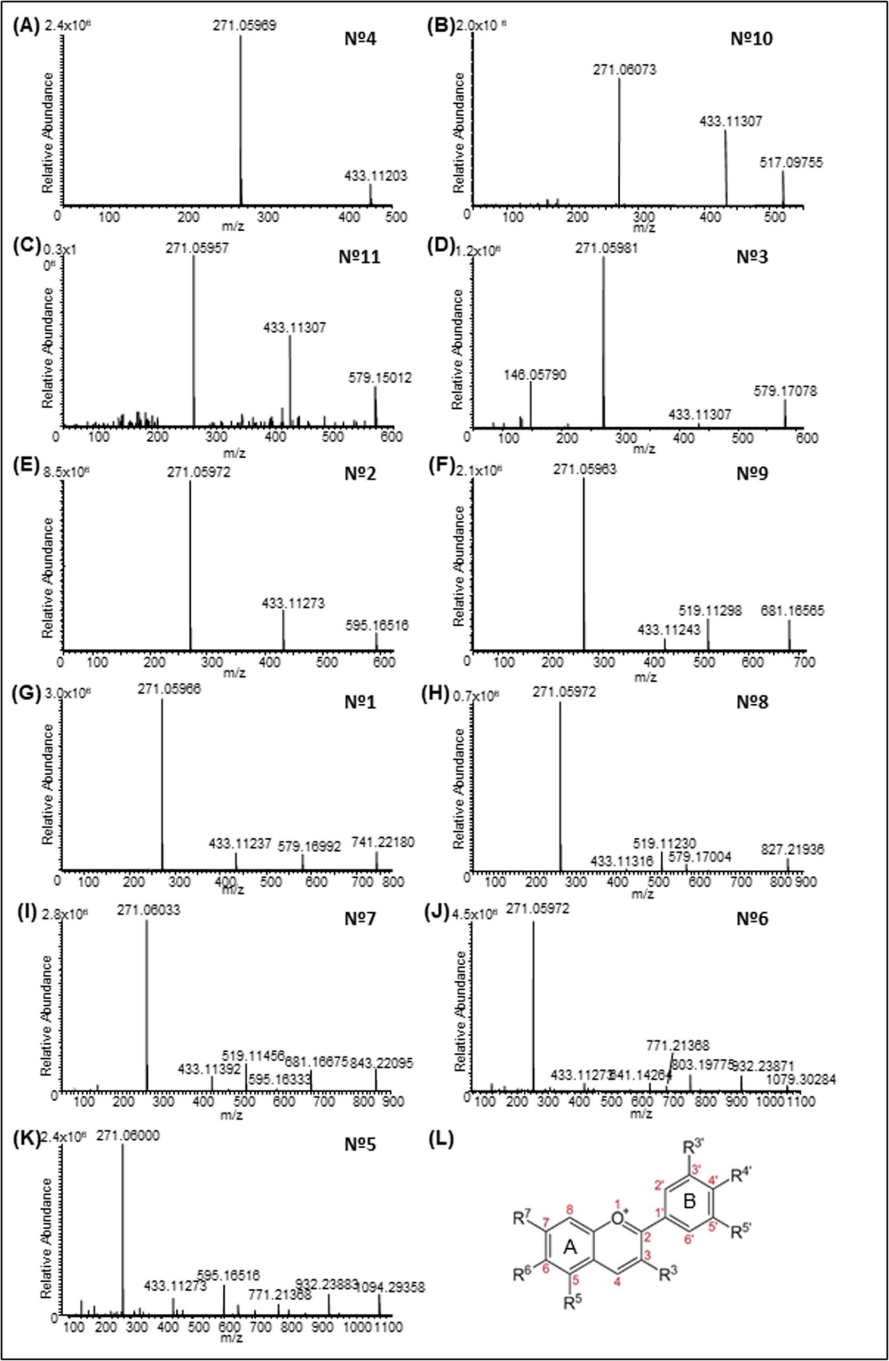
ESI-MS/MS spectra of anthocyanins isolated from petals of *Gentiana lutea* L. var. *aurantiaca*. The number from 1 to 11 corresponded with the anthocyanins characterized and listed in Table 1. The image in L corresponded to the basic structure of the anthocyanin molecule.

All the detected peaks showed a maximum absorbance between 495–505 nm (S1 Fig), characteristic of pelargonidin derivatives [36]. The glycosidic substitution pattern of anthocyanins can be inferred by the presence of absorption in the 400–460 nm region, and the sugar substitution in the 3-OH is deduced by ratio of E440/Emax about two times greater than those for sugar substitution in both 3- and 5-OH positions [37]. The E440/Emax ratios obtained for peak 2 was 25% while peak 4 showed a E440/Emax of 52%, providing evidence that pigment 2 was a 3,5-glycoside and the latter was a 3-glycoside. Identification and peak assignment of anthocyanins was based on the analysis of the mass fragmentation pattern, followed by the comparison of their retention times and mass spectral data with those of standards, if available, and literature data. All the glycosides were tentatively identified using the molecular weight of the aglycone pelargonidin, *m/z* 271.06008 (Fig 2). In total, eleven major pelargonidin derivatives were identified in the petals at different developmental stages (Table 1). The anthocyanins identified were mainly 3-*O*-glycoside conjugates and their derivatives. Peak 1 had *m/z* of 741.22180 and three mass fragment ions at *m/z* 579.16992, due to the elimination of one hexose molecule; at *m/z* 433.11237, indicating the loss of one molecule of rhamnose, and 271.05966, corresponding to pelargonidin (Fig 2G). Therefore, peak 1 was assigned to pelargonidin 3-*O*-rutinoside-5-*O*-β-D-glucoside (C_33_H_41_O_19_) [38]. Peak 2 (Fig 2E) showed a molecular ion at *m/z* 595.16516, and two fragment ions of 433.11273 and 271.05972, respectively. Peak 2 was identified as pelargonidin 3,5-*O*-diglucoside (C_27_H_31_O_15_) [39]. Peak 3 had a molecular ion at *m/z* 579.17078, and three mass fragment ions, one at *m/z* 433.11307, due to the elimination of one molecule of rhamnose; one at *m/z* 271.05981, indicating the loss of rutinoside; and one at 146.05790 for rhamnose. Thus, peak 3 was assigned to pelargonidin 3-*O*-rutinoside (C_27_H_31_O_14_; Fig 2D). Peak 4 displayed at *m/z* 433.11203 and a main mass fragment ion at *m/z* 271.05969, which corresponded to the pelargonidin molecule, due to the elimination of one molecule of glucose (Fig 2A). Therefore, the peak was identified as pelargonidin 3-*O*-glucoside (C_21_H_21_O_10_; Fig 2A). Peak 5 was characterized by a molecular ion *m/z* 1094.29358, and four fragment ions: *m/z* 932.23883, which indicated loss of one hexose; *m/z* 771.21368, indicative of the loss of caffeoylglucoside; *m/z* 433.11273, corresponding to pelargonidin and one glucose molecule; and *m*/z 271.06000, the pelargonidin aglycone. For this reason, peak 5 was designed as pelargonidin 3-*O*-[2-*O*-(6-(E)-feruloyl-β-D-glucopyranosyl)-6-*O*-(E)-caffeoyl-β-D-glucopyranoside]-5-*O*-(β-D-glucopyranoside) (C_52_H_55_O_26_; Fig 2K). Peak 6 had a molecular ion *m/z* 1079.30284, and four fragment ions: *m/z* 932.23871 and *m/z* 771.21368 indicated the loss of coumaroylglucoside; *m/z* 771.21368 and *m/z* 433.11273 indicated the loss of feruloylglucoside, which resulted in pelargonidin glucoside; and *m/z* 271.05972, corresponding to pelargonidin (Fig 2J). Thus, peak 6 was tentatively assigned to pelargonidin 3-*O*-[2-*O*-(6-(E)-feruloyl-β-D-glucopyranosyl)-6-*O*-(E)-*p*-coumaroyl-β-D-glucopyranoside]-5-*O*-(β-D-glucopyranoside) (C_52_H_55_O_25_). Peak 7 showed a *m/z* 843.22095 and five mass fragment ions at *m/*z 681.16675, due to the loss of a caffeic acid; *m/z* 595.16333 [M–248.05321 (malonylhexose)]; *m/z* 519.11456 [M-324.08451 (caffeoylhexose)]; *m/z* 433.11392, corresponding to pelargonidin with one glucose; and, finally, *m/z* 271.06033, the pelargonidin aglycone. This peak (Fig 2I) was tentatively assigned to pelargonidin 3-*O*-(6-*O*-caffeoyl-D-glucoside)-5-*O*-(6-*O*-malonyl-β-D-glucoside) (C_39_H_39_O_21_) [40]. Peak 8 displayed *m/z* 827.21936, and three mass fragment ions at 579.17004 and 519.11230, which corresponded to the loss of malonoylglucoside and coumarylglucoside, respectively; and 271.05972, which represented pelargonidin. Peak 8 was assigned to pelargonidin 3-*O*-(6-*p*-coumaroyl-D-glucoside)-5-(4-*O*-malonyl-β-D-glucoside) (C_36_H_43_O_22_; Fig 2H). Peak 9 was identified as pelargonidin 3-*O*-(6-*O*-malonyl-β-D-glucoside)-5-β-D-glucoside (C_30_H_33_O_18_) on the basis of full MS at *m/z* 681.16565 which, upon the loss of a glucose molecule, showed a fragment of *m/z* 519.11298; and the loss of a malonoylglucoside yielded a fragment at *m/z* 433.11243, corresponding to the pelargonidin unit and a glucose molecule (Fig 2F). Peak 10 and peak 11, finally, showed a molecular ions at *m/z* of 517.09755 and 579.15012, respectively, and were tentatively assigned to pelargonidin 3-*O*-(6-*O*-malonyl-β-D-glucoside) (C_24_H_21_O_13_; Fig 2B) and pelargonidin 3-*O*-(6-*p*-coumaroyl)glucoside (C_30_H_27_O_12_; Fig 2C).

**Table 1:** Identification of anthocyanins from *Gentiana lutea* L. var. *aurantiaca* petals by HPLC-ESI-MS/MS.

Peak number	Rt (min)	UV-maxima (nm)	Experimental [M]^+^ (*m/z*)	Experimental MS/MS (*m/z*)	Formula	Assignment	Petal Stage
**1**	6.88	274, 426, 498	741.22180	579.16992/433.11237/271.05966	C_33_H_41_O_19_	Pelargonidin 3-*O*-rutinoside-5-*O*-β-D-glucoside	S3, S5
**2**	8.61	282, 497	595.16516	433.11273/271.05972	C_27_H_31_O_15_	Pelargonidin 3,5-*O*-diglucoside	S1, S3, S5
**3**	11.15	498	579.17078	433.11307/271.05981/146.05790	C_27_H_31_O_14_	Pelargonidin 3-*O*-rutinoside	S1, S3, S5
**4**	12.53	498	433.11203	271.05969	C_21_H_21_O_10_	Pelargonidin 3-*O*-glucoside	S1, S3, S5
**5**	12.73	282, 496	1094.29358	932.23883/771.21368/433.11273/271.06000	C_52_H_55_O_26_	Pelargonidin 3-*O*-[2-*O*-(6-(E)-feruloyl-β-D-glucopyranosyl)-6-*O*-(E)-caffeoyl-β-D-glucopyranoside]-5-*O*-(β-D-glucopyranoside)	S3, S5
**6**	13.07	447, 496	1079.30284	932.23871/771.21368/433.11273/271.05972	C_52_H_55_O_25_	Pelargonidin 3-*O*-[2-*O*-(6-(E)-feruloyl-β-D-glucopyranosyl)-6-*O*-(E)-*p*-coumaroyl-β-D-glucopyranoside]-5-*O*-(β-D-glucopyranoside)	S3, S5
**7**	13.87	275,366, 500	843.22095	681.16675/595.16333/519.11456/433.11392/271.06033	C_39_H_39_O_21_	Pelargonidin 3-*O*-(6-*O*-caffeoyl-D-glucoside)-5-*O*-(6-*O*-malonyl-β-D-glucoside)	S3, S5
**8**	14.28	492	827.21936	579.17004/519.11230/271.05972	C_36_H_43_O_22_	Pelargonidin 3-*O*-(6-*p*-coumaroyl-D-glucoside)-5-(4-*O*-malonyl-β-D-glucoside)	S3, S5
**9**	14.94	447, 496	681.16565	519.11298/433.11243/271.05963	C_30_H_33_O_18_	Pelargonidin 3-*O*-(6-*O*-malonyl-β-D-glucoside)-5-β-D-glucoside	S3, S5
**10**	15.22	282, 496	517.09755	433.11307/271.06073	C_24_H_21_O_13_	Pelargonidin 3-*O*-(6-*O*-malonyl-β-D-glucoside)	S1, S3, S5
**11**	15.66	447, 496	579.15012	433.11307/271.05957	C_30_H_27_O_12_	Pelargonidin 3-*O*-(6-*p*-coumaroyl)glucoside	S1, S3, S5

### Quantification of pelargonidin derivatives and precursors in petals at three developmental stages

**Fig 3:**
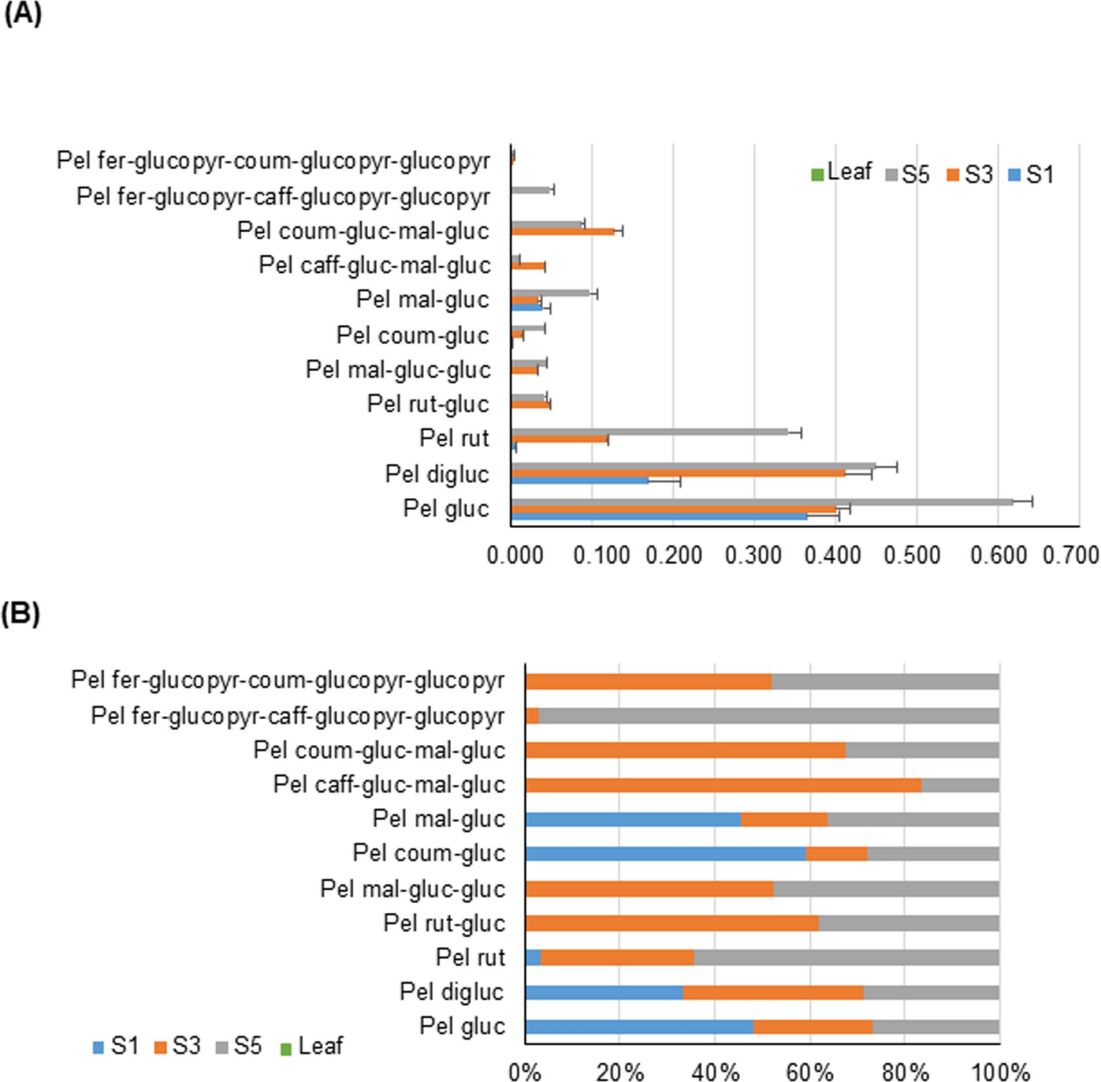
Levels of pelargonidin derivatives identified in leaf and petals of *Gentiana lutea* L. var. *aurantiaca* in three different developmental stages by HPLC-ESI-MS/MS analyses. (A) Data are expressed as average and standard deviation which represent, for each anthocyanin metabolite, the fold over the internal standard (IS), have been obtained by using, at least, 3 independent biological replicates. For more details, see Materials and Methods. (B) Percent relative abundance of each anthocyanin among the three developmental stages (S1, S3, S5) under study. Abbreviations: Pel coum-gluc, Pelargonidin 3-*O*-(6-*p*-coumaroyl)glucoside; Pel digluc, Pelargonidin 3,5-*O*-diglucoside; Pel caff-gluc-mal-gluc, Pelargonidin 3-*O*-(6-*O*-caffeoyl-D-glucoside)-5-*O*-(6-*O*-malonyl-β-D-glucoside); Pel fer-glucopyr-caff-glucopyr-glucopyr, Pelargonidin 3-*O*-[2-*O*-(6-(E)-feruloyl-β-D-glucopyranosyl)-6-*O*-(E)-caffeoyl-β-D-glucopyranoside]-5-*O*-(β-D-glucopyranoside); Pel fer-glucopyr-coum-glucopyr-glucopyr, Pelargonidin 3-*O*-[2-*O*-(6-(E)-feruloyl-β-D-glucopyranosyl)-6-*O*-(E)-*p*-coumaroyl-β-D-glucopyranoside]-5-*O*-(β-D-glucopyranoside); Pel gluc, Pelargonidin 3-*O*-glucoside; Pel mal-gluc, Pelargonidin 3-*O*-(6-*O*-malonyl-β-D-glucoside); Pel rut, Pelargonidin 3-*O*-rutinoside; Pel rut-gluc, Pelargonidin 3-*O*-rutinoside-5-*O*-β-D-glucoside; Pel mal-gluc-gluc, Pelargonidin 3-*O*-(6-*O*-malonyl-β-D-glucoside)-5-β-D-glucoside; Pel coum-gluc-mal-gluc, Pelargonidin 3-*O*-(6-*p*-coumaroyl-D-glucoside)-5-(4-*O*-malonyl-β-D-glucoside).

The anthocyanins determined by HPLC-ESI-MS/MS analyses in the petals of *G*. *lutea* L. var. *aurantiaca* at three developmental stages showed strong variation in abundance (Fig 3). Pelargonidin 3-*O*-glucoside and pelargonidin 3,5-*O*-diglucoside were found to be, by far, the predominant anthocyanins in all the samples analyzed. Interestingly, none of pelargonidin glycosides were detectable in leaves of *G*. *lutea* L. var. *aurantiaca* (Fig 3). Petals at stage one (S1) were green, and in this stage pelargonidin 3-*O*-glucoside (62.5%) and pelargonidin 3,5-*O*-diglucoside (29.1%) were the main anthocyanins detected (Fig 3 and S2 Fig), followed by far by pelargonidin 3-*O*-(6-*O*-malonyl-β-D-glucoside) (6.8%). Other pelargonidin derivatives identified, albeit at very low amounts, were pelargonidin 3-*O*-rutinoside and pelargonidin 3-*O*-(6-*p*-coumaroyl)glucoside. Stage 3 (S3) displayed the presence of new pelargonidin derivatives not detected at S1, with four main derivatives: pelargonidin 3,5-*O*-diglucoside (33.4%), pelargonidin 3-*O*-glucoside (32.5%), pelargonidin-3-*O*-(6-*p*-coumaryl-D-glucoside)-5-(4-*O*-malonyl-β-D-glucoside) (10.3%) and pelargonidin 3-*O*-rutinoside (9.6%) (Fig 3 and S2 Fig). Stage 5 (S5), corresponding to the flower in anthesis, showed the highest content in anthocyanins. In this tissue, the major derivatives of pelargonidin corresponded to pelargonidin 3-*O*-glucoside (34.5%), followed by pelargonidin 3,5-*O*-diglucoside (25.1%), pelargonidin 3-*O*-rutinoside (19.10%) and pelargonidin 3-*O*-(6-*O*-malonyl-β-D-glucoside) (5.5%) (Fig 3). The formation of pelargonidin 3-*O*-glucoside, pelargonidin 5-*O*-glucoside, pelargonidin 3,5-*O*-diglucoside and pelargonidin 3-*O*-rutinoside are the most likely first steps of glycosylation in gentian petals (Fig 1), and are further modified by acylation reactions, leading to more diverse and complex pelargonidin molecules [41]. Notably, the anthocyanins molecules modified with aromatic acyl groups in their 3- and/or 5-positions appear to provide a reddish-purple color (without the interaction with co-pigments and/or metal ions) [41,42]. Overall, the biochemical analyses showed that there was a significant increase in pelargonidin glycosides as the petals developed, and such accumulation parallels the increasing orange coloration of the petals during development. In most plants, total anthocyanins content increases with the flower development and reaches its peak just before the buds open [43,44], with a rapid accumulation of anthocyanins in petals at the later stages of their development [45,46]. In order to better understand the relationships among the different anthocyanins detected, we performed a row-directed hierarchical clustering analysis (HCL) (S2 Fig). This analysis evidenced the presence of a main cluster placed in the top region and composed by pelargonidin 3-*O*-rutinoside and pelargonidin 3-*O*-glucoside, and some of their direct derivatives as pelargonidin 3-*O-*(6-*p*-coumaroyl)glucoside, pelargonidin 3-*O-*(6-*p*-coumaroyl-D-glucoside)-5-(4-*O*-malonyl-β-D-glucoside), and pelargonidin 3-*O*-(6-*O*-malonyl-β-D-glucoside), thus partially confirming the hypothesis that anthocyanins with simpler compositions as pelargonidin 3-*O*-glucoside acts as starting block for the production of more complex derivatives.

**Fig 4:**
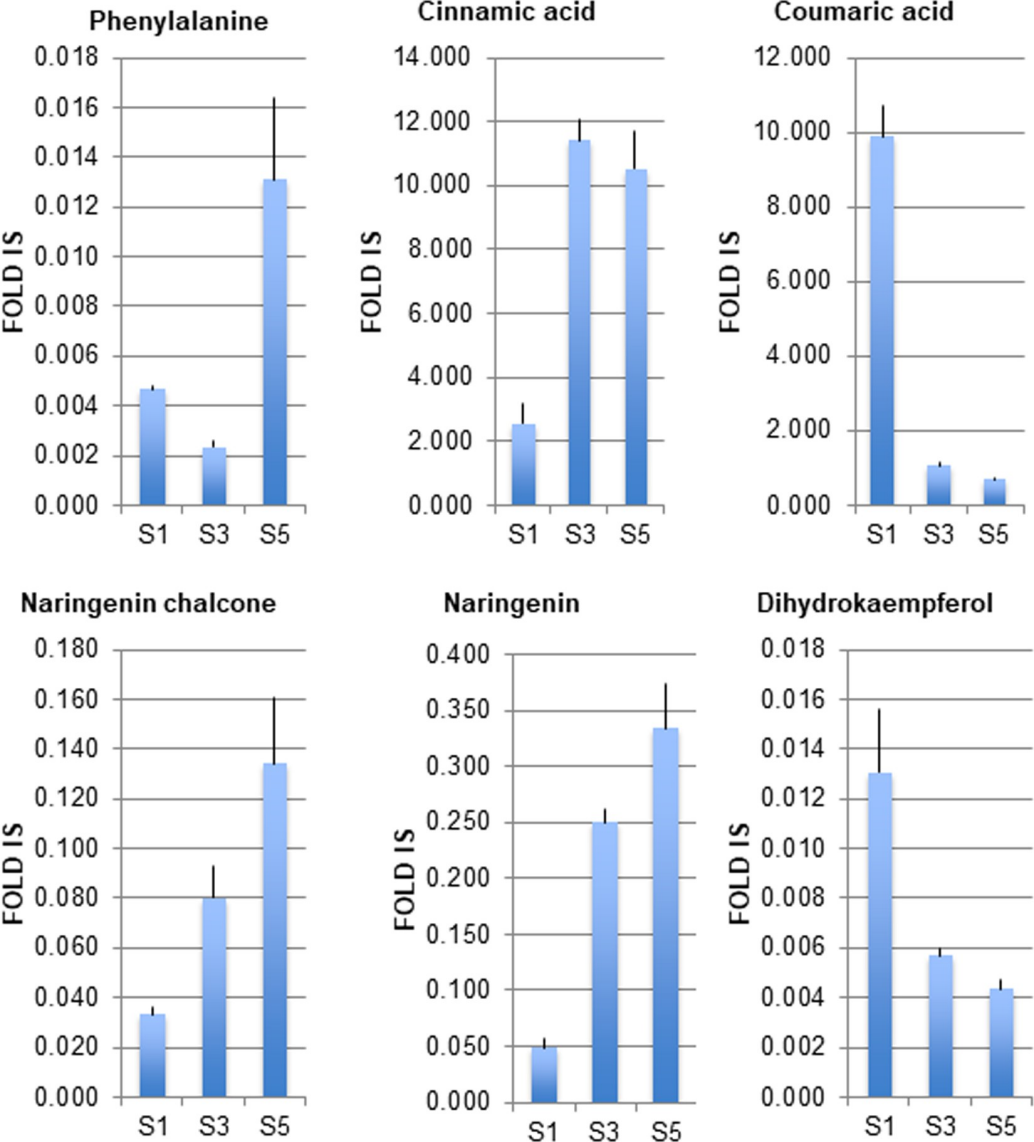
Levels of anthocyanin precursors identified in petals of *Gentiana lutea* L. var. *aurantiaca* in three different developmental stages by HPLC-ESI-MS/MS analyses. Data are expressed as average and standard deviation which represent, for each metabolite, the fold over the internal standard (IS), have been obtained by using, at least, three independent biological replicates. For more details, see Materials and Methods.

We further analyzed the levels of the flavonoid precursors in the anthocyanin pathway in the three developmental stages under study (Fig 4). Phenylalanine acts as the universal precursor of the phenylpropanoid pathway, leading to the production of coumarins, hydroxycinnamic acids, lignins, flavonoids, isoflavonoids, stilbenes, and of a wide variety of other phenolic compounds [6,7]. The levels of phenylalanine decreased from S1 to S3, and reached the maximum levels at S5 stage (Fig 4). Simultaneously, cinnamic acid displayed higher levels in stages S3 and S5, a pattern opposite to that of coumaric acid (Fig 4), which showed its highest content at S1 stage; finally late anthocyanins precursors like naringenin chalcone and naringenin gradually increased from S1 to S5 stages, while dihydrokaempferol was greatly reduced during flower development. The divergent trends of accumulation between a first metabolic group composed by cinnamic acid, naringenin chalcone and naringenin (increased levels from S1 to S5), and a second including coumaric acid and dihydrokaempferol (higher levels at S1, and lower at S3 and S5), could suggest the presence of a series of rate-limiting steps in gentian phenylpropanoid pathway, like in the case of cinnamic acid 4-hydroxylase (C4H) catalyzing aromatic ring-4 hydroxylation of cinnamic acid into *p*-coumaric acid, and/or flavonoid 3-hydroxylase (F3H) utilizing naringenin to yield dihydrokaempferol, which constrain pelargonidin accumulation in the petals during their development. In agreement with this hypothesis, PAL (phenylalanine ammonia-lyase) and C4H are part of a feedback loop controlling phenylpropanoid biosynthesis [47], which is acting at the entry point of the pathway; while F3H plays a role in regulating anthocyanin production in *Antirrhinum* [48].

### Pelargonidin glucoside biosynthesis gene expression

**Fig 5:**
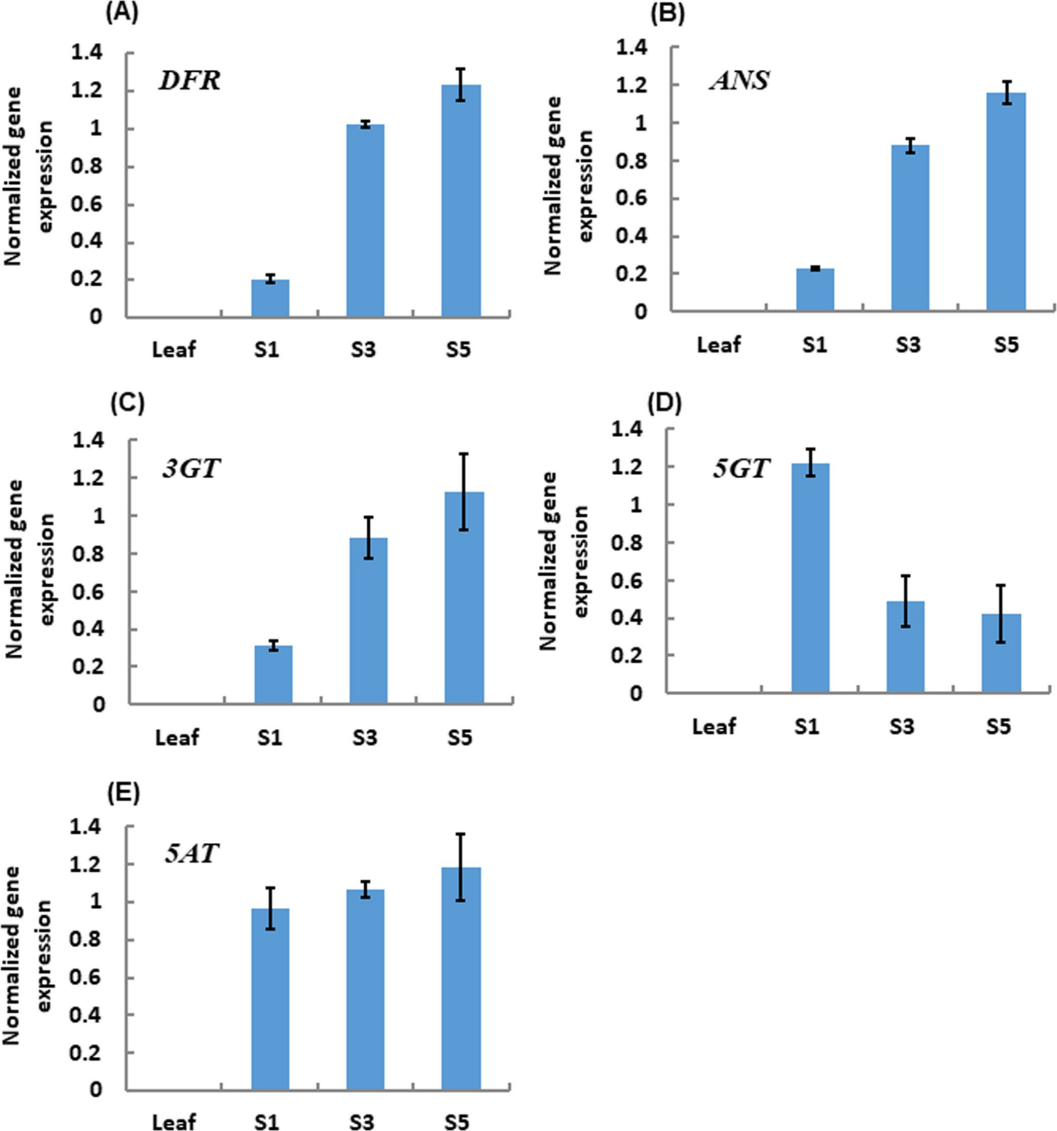
Quantitative expression of anthocyanin genes, normalized on the ubiquitin housekeeping gene in leaf and petals of *Gentiana lutea* L. var. *aurantiaca*. qRT-PCT data were calculated from three biological replicates with at least three technical replicates for each biological replicate and with error bars representing the standard deviation. Abbreviations: *5AT*, *anthocyanin 5-aromatic acyltransferase gene*; *ANS*, *anthocyanidin synthase* gene; *DFR*, *dihydroflavonol 4-reductase* gene; *3GT*, *UDP-glucose*:*flavonoid 3-O-glucosyltransferase* gene; *5GT*, *UDP-glucose*:*flavonoid 5-O-glucosyltransferase* gene.

As anthocyanin genes are mainly regulated at the level of transcription [49], we analyzed the expression levels of five key late anthocyanin biosynthesis genes (*DFR*, *ANS*, *3GT*, *5GT* and *5AT*) involved in the pelargonidin glycoside biosynthesis (Fig 1) in mature leaves and petals of *G*. *lutea* L. var. *aurantiaca* at S1, S3 and S5 stages. *DFR*, *ANS* and *3GT* genes were isolated in a recent study [21]. Furthermore, we cloned *5GT* and *5AT* gene fragments from mixed petals of stages S1, S3 and S5 by RT-PCR (see Material and Methods). Each PCR yielded a single product of the expected size. Cloning, sequencing and alignments of these cDNA fragments and their deduced encoding proteins (S3 and S4 Figs) demonstrated the isolated *5GT* and *5AT* partial cDNAs had high identity (more than 88.0%, eliminating primer sequences) with those from *G*. *triflora* [10]. Quantitative RT-PCR (qRT-PCR) results of anthocyanin genes of interest are reported in Fig 5. The petals of *G*. *lutea* L. var. *aurantiaca* contained substantially higher levels of *DFR*, *ANS*, *3GT*, *5GT* and *5AT* mRNAs, while leaves displayed no accumulation of these mRNAs. In greater detail, higher relative levels of *DFR*, *ANS* and *3GT* mRNAs at stages S3 and S5 than at stage S1 were found, while similar levels of *5AT* mRNA at stages S1, S3 and S5 were observed (Fig 5). These findings are in agreement with previous studies, which showed relatively high expression levels of acyltransferase genes in the initial stages of petal development [50]. Notably, the levels of *5GT* mRNA at stage S1 were higher than at stages S3 and S5. A similar phenomenon has already been observed in carnation [51] and peony [52], where the gene encoding 5GT is also expressed independently of other genes for anthocyanin biosynthesis [53].

### Integration of transcript and metabolite data of gentian phenylpropanoid pathway

**Fig 6:**
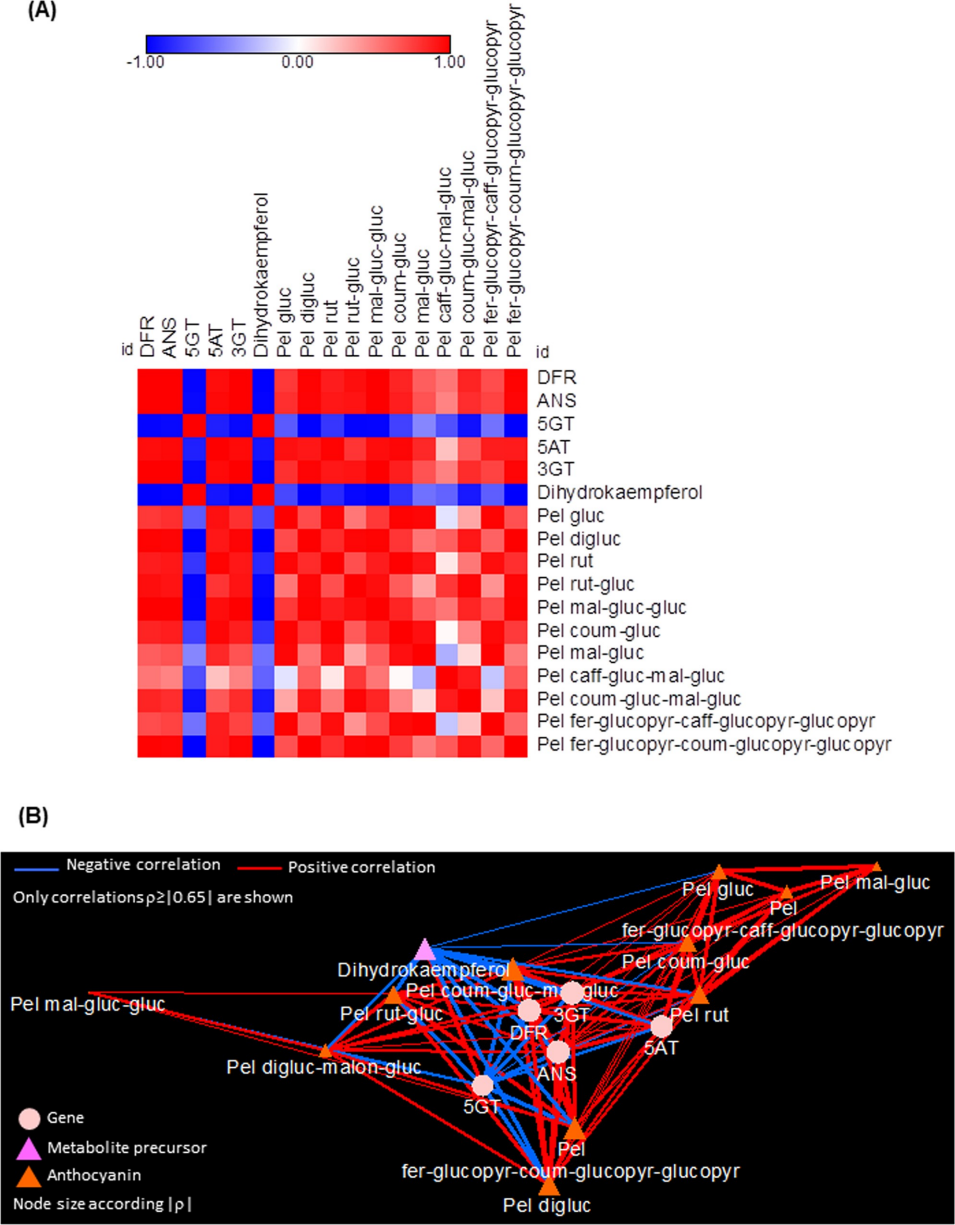
Integration of transcript-metabolite data involved in *G*. *lutea* L. var. *aurantiaca* anthocyanin metabolism. (A) Pearson coefficient-based correlation matrix. Legend on the right corresponds to the names of the anthocyanin transcripts and metabolites. Red and blue shaded boxes represent different levels of positive and negative correlations, respectively; white boxes represent no correlation. (B) Anthocyanin transcript/metabolite correlation network using a prefuse force-directed layout (only ρ > 0.65 are shown). Transcripts, anthocyanins and anthocyanin precursors are represented, respectively, as pink rounds, and orange and violet triangles. Blue and red edges refer to negative and positive correlations, respectively. Node size is according to the node strength (ns, representing the average of all the ρs of each node). Lines joining the nodes indicate positive (red) and negative (blue) correlations, of width proportional to each corresponding |ρ|. Abbreviations: ANS, anthocyanidin synthase; 5AT, anthocyanin 5-aromatic acyltransferase; DFR, dihydroflavonol 4-reductase; 3GT, UDP-glucose:flavonoid 3-*O*-glucosyltransferase gene; 5GT, UDP-glucose:flavonoid 5-*O*-glucosyltransferase; Pel coum-gluc, Pelargonidin 3-*O*-(6-*p*-coumaroyl)glucoside; Pel digluc, Pelargonidin 3,5-*O*-diglucoside; Pel caff-gluc-mal-gluc, Pelargonidin 3-*O*-(6-*O*-caffeoyl-D-glucoside)-5-*O*-(6-*O*-malonyl-β-D-glucoside); Pel fer-glucopyr-caff-glucopyr-glucopyr, Pelargonidin 3-*O*-[2-*O*-(6-(E)-feruloyl-β-D-glucopyranosyl)-6-*O*-(E)-caffeoyl-β-D-glucopyranoside]-5-*O*-(β-D-glucopyranoside); Pel fer-glucopyr-coum-glucopyr-glucopyr, Pelargonidin 3-*O*-[2-*O*-(6-(E)-feruloyl-β-D-glucopyranosyl)-6-*O*-(E)-*p*-coumaroyl-β-D-glucopyranoside]-5-*O*-(β-D-glucopyranoside); Pel gluc, Pelargonidin 3-*O*-glucoside; Pel mal-gluc, Pelargonidin 3-*O*-(6-*O*-malonyl-β-D-glucoside); Pel rut, Pelargonidin 3-*O*-rutinoside; Pel rut-gluc, Pelargonidin 3-*O*-rutinoside-5-*O*-β-D-glucoside; Pel mal-gluc-gluc, Pelargonidin 3-*O*-(6-*O*-malonyl-β-D-glucoside)-5-β-D-glucoside; Pel coum-gluc-mal-gluc, Pelargonidin 3-*O*-(6-*p*-coumaroyl-D-glucoside)-5-(4-*O*-malonyl-β-D-glucoside).

To investigate more in the detail gentian anthocyanin biosynthesis, we calculated Pearson correlation coefficients (ρs) for each data pair (transcript-transcript, transcript-metabolite, metabolite-metabolite), followed by correlation matrix and network visualizations (Fig 6A and 6B, respectively; see Materials and Methods). Overall, most of the correlations observed were of positive sign and very strong (ρ≥0.65), indicating that most of transcript and metabolite levels are showing similar trends in their expression and accumulation. Thus, although at mathematical-based degree, this finding could suggest the presence of a high extent of coordination in the synthesis and the accumulation of pelargonidin anthocyanins along the flower petal development. However, a notable exception was found within *5GT* transcript (Fig 6A). The presence of a divergent altitude for this element could be associated to specific controls at gene expression or metabolite levels (rate limiting steps or negative feedback, as mentioned before). In addition, force-directed correlation network was exploited to highlight the most relevant elements in the dataset (“hubs” of the network) mainly responsible for the anthocyanin production and accumulation (Fig 6B). Interestingly, most of the pelargonidin anthocyanins placed, in a right- and top-side region in the network. Since, in a correlation network, the topology is generated by the correlations of the input file themselves, and nodes which do not display high number of significant correlations towards the rest of the nodes in the dataset normally localize in distal regions of the network, this evidence could provide clues about a non-active role in the control of the pathway. On the other hand, we found most of the genes located in the central area of the network. Among them, *DFR*, *ANS* and *3GT* were the ones displaying the highest number of very significant correlations, as evidenced by their topology as well by the node strength (ns) values (e.g. the average of all the |ρs| yielded by each node) (S1 Table). Finally, the determination of a such high network strength (NS; equal to 0.76) obtained by through the calculation of all the |ρs| in the network, could confirm the existence of strong and harmonic relationships both at transcript and metabolite level, underlying the generation of all the anthocyanin set in the petals of the orange gentian.

## Conclusions

Pelargonidin derivatives have been found in different plant species including orange cultivars of *Pelargonium* where they can account for up to 98% of total anthocyanins [54], *Dahlia variabilis* [55], magenta flowers of *Verbena* [56], red-purple flowers of *Matthiola incana* [57] and *Ipomoea purpurea* [58], red flowers of *Hyacinthus orientalis* [40], maroon flowers of *Pharbitis nil* [59], and white and pink flowers of *Muscari* spp. [44]. All identified anthocyanin molecules of *G*. *lutea* L. var *aurantiaca* were derived from side group modifications of pelargonidin, through different mechanisms of glycosylation and acylation. These modifications have been reported to increase anthocyanin stability in aqueous solution and may likely alter their light absorption properties [6,60]. All metabolites identified contained at least one sugar group. The hydroxyl groups at C_3_ and C_5_ positions of pelargonidin have been reported to be the two commonest targets of glucosylation [60]. In the present study, we revealed that pelargonidin 3-*O*-glucoside, pelargonidin 3,5-*O*-diglucoside and pelargonidin 3-*O*-rutinoside are the main pelargonidin derivatives present in *G*. *lutea* L. var *aurantiaca* petals. However, besides the presence of these molecules, other more complex pelargonidin derivatives were tentatively identified. We analyzed the expression of late *DFR*, *ANS*, *3GT*, *5GT* and *5AT* genes specifically for anthocyanin biosynthesis in the petals of *G*. *lutea* L. var *aurantiaca* to determine the molecular mechanisms responsible for the accumulation of pelargonidin glycosides during flower development. The accumulation of the pelargonidin glucosides parallels the expression levels of the genes *DFR*, *ANS* and *3GT*, suggesting their main role in the accumulation of these anthocyanins in *G*. *lutea* L. var *aurantiaca* flowers, with DFR most probably acting as the limiting step in this process [21]. The resulting data will enhance our understanding of the structural diversity of anthocyanin molecules in *Gentiana* species, and lays a foundation for breeding of flower color and genetic variation studies on *Gentiana* varieties.

## Supporting information

S1 Fig HPLC-PDA chromatographic profiles of the anthocyanins in *Gentiana lutea* L. var. *aurantiaca* petals at the S5 stage. A, HPLC-DAD/UV isoplot from 200–600 nm. Anthocyanins are detected in the 500 nm range. B and C, correspond to magnifications in the 500 nm range to show up the anthocyanins present in lower amounts. D, HPLC-DAD chromatogram at 505 nm, showing the profile of the anthocyanins present in the S5 extract.
(PDF)

S2 Fig Row-directed Hierarchical clustering (HCL) visualization of anthocyanin metabolites detected in petals of *G*. *lutea* L. var. *aurantiaca*. Data represent, for each metabolite, the fold over the internal standard (IS) intensity. Abbreviations: Pel coum-gluc, Pelargonidin 3-*O*-(6-*p*-coumaroyl)glucoside; Pel digluc, Pelargonidin 3,5-*O*-diglucoside; Pel caff-gluc-mal-gluc, Pelargonidin 3-*O*-(6-*O*-caffeoyl-D-glucoside)-5-*O*-(6-*O*-malonyl-β-D-glucoside); Pel fer-glucopyr-caff-glucopyr-glucopyr, Pelargonidin 3-*O*-[2-*O*-(6-(E)-feruloyl-β-D-glucopyranosyl)-6-*O*-(E)-caffeoyl-β-D-glucopyranoside]-5-*O*-(β-D-glucopyranoside); Pel fer-glucopyr-coum-glucopyr-glucopyr, Pelargonidin 3-*O*-[2-*O*-(6-(E)-feruloyl-β-D-glucopyranosyl)-6-*O*-(E)-*p*-coumaroyl-β-D-glucopyranoside]-5-*O*-(β-D-glucopyranoside); Pel gluc, Pelargonidin 3-*O*-glucoside; Pel mal-gluc, Pelargonidin 3-*O*-(6-*O*-malonyl-β-D-glucoside); Pel rut, Pelargonidin 3-*O*-rutinoside; Pel rut-gluc, Pelargonidin 3-*O*-rutinoside-5-*O*-β-D-glucoside; Pel mal-gluc-gluc, Pelargonidin 3-*O*-(6-*O*-malonyl-β-D-glucoside)-5-β-D-glucoside; Pel coum-gluc-mal-gluc, Pelargonidin 3-*O*-(6-*p*-coumaroyl-D-glucoside)-5-(4-*O*-malonyl-β-D-glucoside).
(PDF)

S3 Fig **Alignments of partial *5GT* and *5AT* cDNA sequences encoding UDP-glucose:flavonoid 5-*O*-glucosyltransferase (5GT; A) and anthocyanin 5-aromatic acyltransferase (5AT; B) between *Gentiana triflora* (*Gt*) and *G*. *lutea* L. var. *aurantiaca* (*Gla*).** The underlined cDNA sequences indicate the primers used to isolate cDNA fragments from petals of *G*. *lutea* L. var. *aurantiaca*. Gaps are inserted with a dash (-) in one of the sequences. Abbreviations: Gt, *Gentiana triflora*; Gla, *G*. *lutea* L. var. *aurantiaca*; 5GT, *UDP-glucose*:*flavonoid 5-O-glucosyltransferase* gene; 5AT, *anthocyanin 5-aromatic acyltransferase* gene. GenBank accession numbers: Gt5GT, AB363839; Gt5AT, AB010708. The partial cDNA sequences of *5GT* and *5AT* genes from *G*. *lutea* L. var. *aurantiaca* have been isolated by the authors in this study.
(PDF)

S4 Fig **Alignments of the deduced amino acid sequences encoded by UDP-glucose:flavonoid 5-*O*-glucosyltransferase (5GT) gene (A), and anthocyanin 5-aromatic acyltransferase (5AT) gene (B) from *Gentiana triflora* and *G*. *lutea* L. var. *aurantiaca*.** Gaps are inserted with a dash (-) in one of the sequences. The underlined amino acid sequences from *G*. *lutea* L. var. *aurantiaca* were deduced from primers. Abbreviations: Gt, *Gentiana triflora*; Gla, *G*. *lutea* L. var. *aurantiaca*; 5GT, UDP-glucose:flavonoid 5-*O*-glucosyltransferase; 5AT, anthocyanin 5-aromatic acyltransferase. GenBank accession numbers: *Gt5GT*, AB363839; *Gt5AT*, AB010708. The partial amino acid sequences of 5GT and 5AT of *G*. *lutea* L. var. *aurantiaca* were deduced from partial cDNA sequences cloned by the authors in this study.
(PDF)

S1 Table Node strength (ns) and network strength (NS) of gentian phenylpropanoid genes and metabolites, expressed as the average of all the |ρs| yielded by a node and the average of the ns, respectively. (PDF)
