# Supplementary material for: Differential accumulation of pelargonidin glycosides in petals at three different developmental stages of the orange-flowered gentian (Gentiana lutea L. var. aurantiaca)
Source: PLoS One. 2019 Feb 11;14(2):e0212062. doi: 10.1371/journal.pone.0212062 (PMC6370212; doi:10.1371/journal.pone.0212062)
Supplement: S1 Fig — A, HPLC-DAD/UV isoplot from 200–600 nm. Anthocyanins are detected in the 500 nm range. B and C, correspond to magnifications in the 500 nm range to show up the anthocyanins present in lower amounts. D, HPLC-DAD chromatogram at 505 nm, showing the profile of the anthocyanins present in the S5 extract. (PDF) [file pone.0212062.s001.pdf]

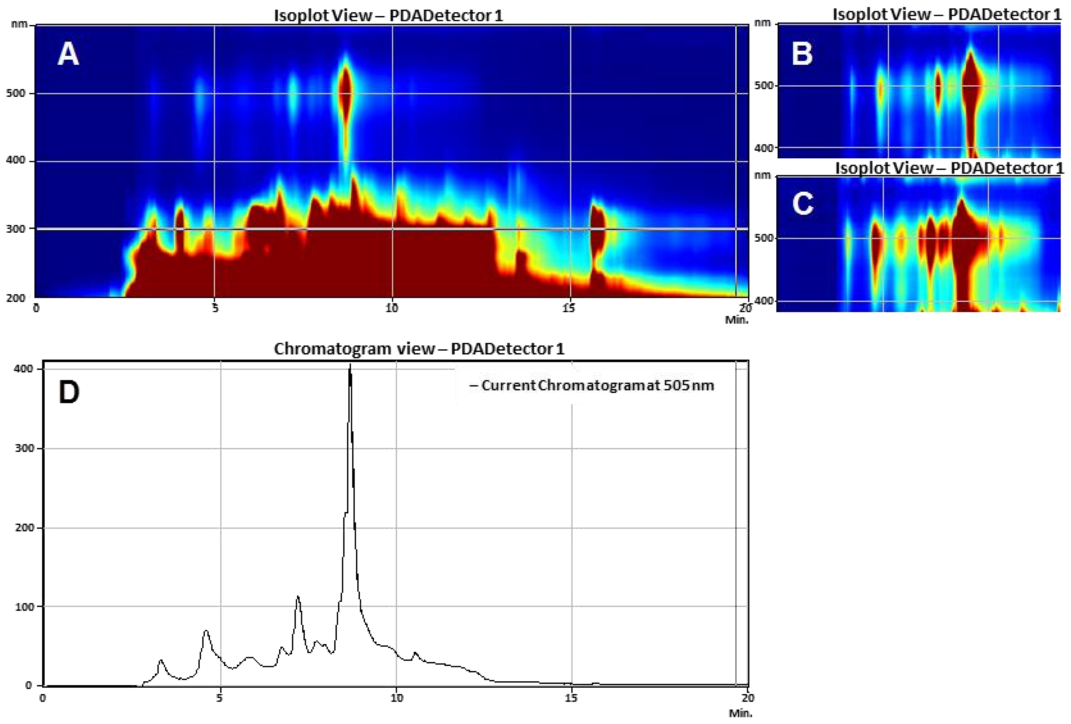

S1 Fig. Typical HPLC-DAD/UV isoplot chromatograms and HPLC-PDA chromatographic profile of the anthocyanins in *Gentiana lutea* L. var. *aurantiaca* petals at the S5 stage of development. A) HPLC-DAD/UV isoplot from 200-600 nm. Anthocyanins are detected in the 500 nm range. B) and C) correspond to magnifications in the 500 nm range to show up the anthocyanins present in lower amounts. D) HPLC-DAD chromatogram at 505 nm, showing the profile of the anthocyanins present in the S5 extract.
