# Supplementary material for: Differential accumulation of pelargonidin glycosides in petals at three different developmental stages of the orange-flowered gentian (Gentiana lutea L. var. aurantiaca)
Source: PLoS One. 2019 Feb 11;14(2):e0212062. doi: 10.1371/journal.pone.0212062 (PMC6370212; doi:10.1371/journal.pone.0212062)
Supplement: S2 Fig — Data represent, for each metabolite, the fold over the internal standard (IS) intensity. Abbreviations: Pel coum-gluc, Pelargonidin 3-O-(6-p-coumaroyl)glucoside; Pel digluc, Pelargonidin 3,5-O-diglucoside; Pel caff-gluc-mal-gluc, Pelargonidin 3-O-(6-O-caffeoyl-D-glucoside)-5-O-(6-O-malonyl-β-D-glucoside); Pel fer-glucopyr-caff-glucopyr-glucopyr, Pelargonidin 3-O-[2-O-(6-(E)-feruloyl-β-D-glucopyranosyl)-6-O-(E)-caffeoyl-β-D-glucopyranoside]-5-O-(β-D-glucopyranoside); Pel fer-glucopyr-coum-glucopyr-glucopyr, Pelargonidin 3-O-[2-O-(6-(E)-feruloyl-β-D-glucopyranosyl)-6-O-(E)-p-coumaroyl-β-D-glucopyranoside]-5-O-(β-D-glucopyranoside); Pel gluc, Pelargonidin 3-O-glucoside; Pel mal-gluc, Pelargonidin 3-O-(6-O-malonyl-β-D-glucoside); Pel rut, Pelargonidin 3-O-rutinoside; Pel rut-gluc, Pelargonidin 3-O-rutinoside-5-O-β-D-glucoside; Pel mal-gluc-gluc, Pelargonidin 3-O-(6-O-malonyl-β-D-glucoside)-5-β-D-glucoside; Pel coum-gluc-mal-gluc, Pelargonidin 3-O-(6-p-coumaroyl-D-glucoside)-5-(4-O-malonyl-β-D-glucoside). (PDF) [file pone.0212062.s002.pdf]

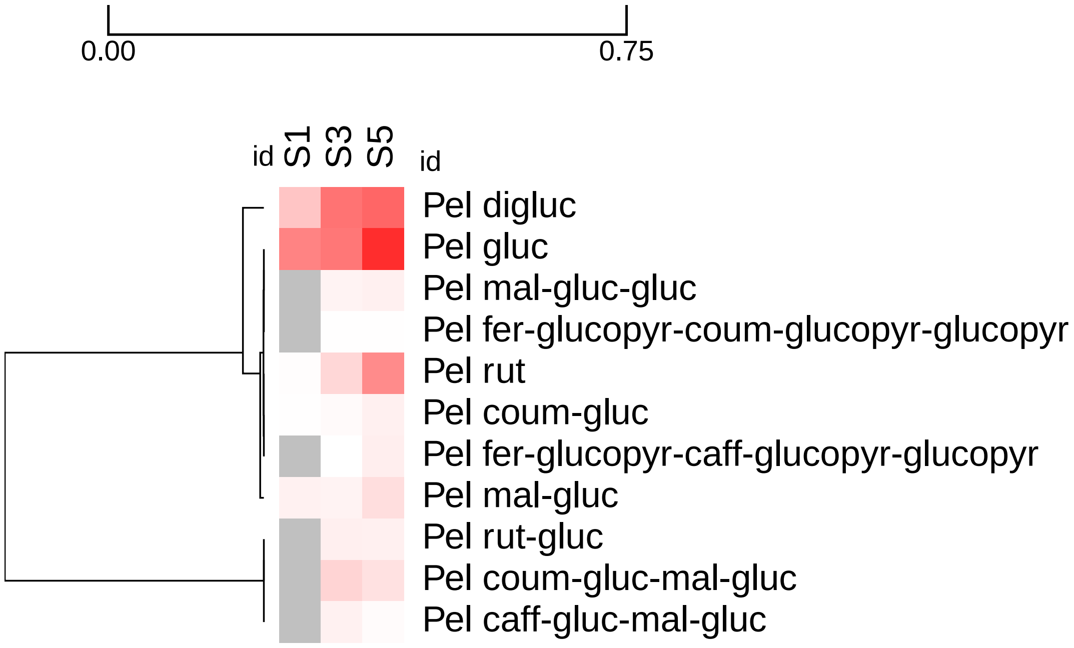

S2 Fig. Row-directed Hierarchical clustering (HCL) visualization of anthocyanin metabolites detected in flower petals of *G. lutea* L. var. *aurantiaca*. Data represent, for each metabolite, the fold over the internal standard (IS) intensity. Abbreviations: Pel coum-gluc, Pelargonidin 3-*O*-(6-*p*-coumaroyl)glucoside; Pel digluc, Pelargonidin 3,5-*O*-diglucoside; Pel caff-gluc-mal-gluc, Pelargonidin 3-*O*-(6-*O*-caffeoyl-D-glucoside)-5-*O*-(6-*O*-malonyl-β-D-glucoside); Pel fer-glucopyr-caff-glucopyr-glucopyr, Pelargonidin 3-*O*-[2-*O*-(6-(E)-feruloyl-β-D-glucopyranosyl)-6-*O*-(E)-caffeoyl-β-D-glucopyranoside]-5-*O*-(β-D-glucopyranoside); Pel fer-glucopyr-coum-glucopyr-glucopyr, Pelargonidin 3-*O*-[2-*O*-(6-(E)-feruloyl-β-D-glucopyranosyl)-6-*O*-(E)-*p*-coumaroyl-β-D-glucopyranoside]-5-*O*-(β-D-glucopyranoside); Pel gluc, Pelargonidin 3-*O*-glucoside; Pel mal-gluc, Pelargonidin 3-*O*-(6-*O*-malonyl-β-D-glucoside); Pel rut, Pelargonidin 3-*O*-rutinoside; Pel rut-gluc, Pelargonidin 3-*O*-rutinoside-5-*O*-β-D-glucoside; Pel mal-gluc-gluc, Pelargonidin 3-*O*-(6-*O*-malonyl-β-D-glucoside)-5-β-D-glucoside; Pel coum-gluc-mal-gluc, Pelargonidin 3-*O*-(6-*p*-coumaroyl-D-glucoside)-5-(4-*O*-malonyl-β-D-glucoside).
