# Supplementary material for: Differential accumulation of pelargonidin glycosides in petals at three different developmental stages of the orange-flowered gentian (Gentiana lutea L. var. aurantiaca)
Source: PLoS One. 2019 Feb 11;14(2):e0212062. doi: 10.1371/journal.pone.0212062 (PMC6370212; doi:10.1371/journal.pone.0212062)
Supplement: S3 Fig — Alignments of partial 5GT and 5AT cDNA sequences encoding UDP-glucose:flavonoid 5-O-glucosyltransferase (5GT; A) and anthocyanin 5-aromatic acyltransferase (5AT; B) between Gentiana triflora (Gt) and G. lutea L. var. aurantiaca (Gla). The underlined cDNA sequences indicate the primers used to isolate cDNA fragments from petals of G. lutea L. var. aurantiaca. Gaps are inserted with a dash (-) in one of the sequences. Abbreviations: Gt, Gentiana triflora; Gla, G. lutea L. var. aurantiaca; 5GT, UDP-glucose:flavonoid 5-O-glucosyltransferase gene; 5AT, anthocyanin 5-aromatic acyltransferase gene. GenBank accession numbers: Gt5GT, AB363839; Gt5AT, AB010708. The partial cDNA sequences of 5GT and 5AT genes from G. lutea L. var. aurantiaca have been isolated by the authors in this study. (PDF) [file pone.0212062.s003.pdf]

A

```
                   551                                               600
    Gt5GT    (551) GAATCCTGTATACTACTCTGCTTCCTTGGGCTGCCGATGTGGCTCGTGAA
   Gla5GT      (1) -----------------CTGCTTCCTTGGGCTGCCGATGTGGCTCGTGAA
Consensus    (551)                  CTGCTTCCTTGGGCTGCCGATGTGGCTCGTGAA
                   601                                               650
    Gt5GT    (601) TTTCGCTTACCCTCTGTGCTTCTGTGGACACAACCAGTCACAACTTTCCT
   Gla5GT     (34) TTTCGCATACCATCGGTACTTCTGTGGACGCAGCCAGTCACAACTTTGGT
Consensus    (601) TTTCGC TACC TC GT CTTCTGTGGAC CA CCAGTCACAACTTT  T
                   651                                               700
    Gt5GT    (651) GACATTCCACTACTATTTCACTGGCTATGAAGATGCTATCAACAAGGTAC
   Gla5GT     (84) GACATTCCACTACTATTTCAGTGGCTATGAAGACGCAATCAAGGAAGTAT
Consensus    (651) GACATTCCACTACTATTTCA TGGCTATGAAGA GC ATCAA  A GTA
                   701                                               750
    Gt5GT    (701) GCAATCAACAAGGTACAGAAGATGATTCCACTATTCAGCTTCCTAGACTT
   Gla5GT    (134) GCAAT------GGTACAG------ATTCAACGATTCAGCTTCCAAGACTT
Consensus    (701) GCAAT      GGTACAG      ATTC AC ATTCAGCTTCC AGACTT
                   751                                               800
    Gt5GT    (751) CCCCTGCTGTCTAGTCGTGATTTACATTCTTTCATGCTACCATCTAATCC
   Gla5GT    (172) CCCCTGCTGTCTAGCCGGGATCTTCATTCTTTCATGCTTCCGTCAAATCC
Consensus    (751) CCCCTGCTGTCTAG CG GAT T CATTCTTTCATGCT CC TC AATCC
                   801                                               850
    Gt5GT    (801) GTTTAAAGGTGCGATCAACACGTTCAAAGAGCACCTGGAAGCACTTGATG
   Gla5GT    (222) GTTTAAAGGTGCACTCAAGACGTTCAAAGAGCACCTGGAAGCGCTTGACG
Consensus    (801) GTTTAAAGGTGC  TCAA ACGTTCAAAGAGCACCTGGAAGC CTTGA G
                   851                                               900
    Gt5GT    (851) CAGAAGAAACTCCTCCAACCATTCTTGTTAACAGTTACGATGCGTTAGAG
   Gla5GT    (272) CAGAACAAAATCCGACAA---TTATTGTTAACAGTTACGACGCTTTAGAG
Consensus    (851) CAGAA AAA TCC  CAA   TT TTGTTAACAGTTACGA GC TTAGAG
                   901                                               950
    Gt5GT    (901) GAAGAGGCCTTGCAGGCGATGATCCCAAAGTACAAAACAATGGGGATCGG
   Gla5GT    (319) GAAGAGGCCTTGCAAGCAAT---CCCAAAGTACAAAACAATGGGGATTGG
Consensus    (901) GAAGAGGCCTTGCA GC AT   CCCAAAGTACAAAACAATGGGGAT GG
                   951                                              1000
    Gt5GT    (951) ACCATTGATTCCCTCGTCCGTTTTCGACACCAGGGAAACAACATGTGAGG
   Gla5GT    (366) ACCACTGATTCCCTCATCCATTTTTGATACCAAGGAAACA---TCTGAGG
Consensus    (951) ACCA TGATTCCCTC TCC TTTT GA ACCA GGAAACA   T TGAGG
                   1001                                             1050
    Gt5GT   (1001) TGGTTTCTCTTGTTCCAGATTTGGCGCAAAAGTCAAAGGACGACTGCCAG
   Gla5GT    (413) T---TTCTCTTGTTGCATATTTGTTGCAAAAGTCTAAGGAC---------
Consensus   (1001) T   TTCTCTTGTT CA ATTTG  GCAAAAGTC AAGGAC
                   1051                                             1100
    Gt5GT   (1051) TGGCATGGATGGTTGAACTCAAAAGCAGAGGGGTCAGTGATTTATGTATC
   Gla5GT    (451) TGCCATGGATGGTTGAACTCAAAACCCCAAGGGTCAGTGATTTATGTATC
Consensus   (1051) TG CATGGATGGTTGAACTCAAAA C  A GGGTCAGTGATTTATGTATC
                   1101                                             1150
    Gt5GT   (1101) ATTCGGAAGTCATGTCAAGCAATCTAAAGCTCAAACGGAGGAGATAGCAA
   Gla5GT    (501) ATTTGGAAGTCATGTGAAGCAGTCTAAATCCCAAACGGAGGAGATAGCAA
Consensus   (1101) ATT GGAAGTCATGT AAGCA TCTAAA C CAAACGGAGGAGATAGCAA
                   1151                                             1200
    Gt5GT   (1151) AAGGGCTCCTCGCGAGTGGCCATCCATTCTTGTGGGTGATTACATCGAAC
   Gla5GT    (551) AAGGGCTCCTCGCGAGTGGCCATCCATTCTTGTGGGTGATTACATCAAAT
Consensus   (1151) AAGGGCTCCTCGCGAGTGGCCATCCATTCTTGTGGGTGATTACATC AA
                   1201                                             1250
    Gt5GT   (1201) GAAGAAGAGG------AGGGTGATGAAATAATGGAACAAAATTTGGTGGA
   Gla5GT    (601) GAGGAGGAGGCCGAAAAAGGAAAAGTACTAATGGAACGAAATTTGCTGAA
Consensus   (1201) GA GA GAGG      A GG  A G A TAATGGAAC AAATTTG TG A
                   1251                                             1300
```

```
    Gt5GT  (1245) GGAAATTCAAGAAAAAGGGATGATGATAGTACCATGGTGTGCTCAGTTTC
   Gla5GT   (651) GGAAATTCAAGAAAAAGGGATGA---TAGTACCATGGTGTGCTCAGATTC
Consensus  (1251) GGAAATTCAAGAAAAAGGGATGA   TAGTACCATGGTGTGCTCAG TTC
                  1301                                          1350
    Gt5GT  (1295) AGGTGCTAAAGCATCCGTCTGTGGGATGCTTCATGACACATTGCGGATGG
   Gla5GT   (698) AGGTGCTAAAGCATCACTCGGTGGGATGTTTCATGACACATTGCGGATGG
Consensus  (1301) AGGTGCTAAAGCATC  TC GTGGGATG TTCATGACACATTGCGGATGG
                  1351                                          1400
    Gt5GT  (1345) AATTCAACGCTGGAGAGCATAGCTTGTGGGGTGCCTATGATTGGTTTTCC
   Gla5GT   (748) AATTCAACGCTGGAGAGC--------------------------------
Consensus  (1351) AATTCAACGCTGGAGAGC
```

B

```
                  451                                            500
    Gt5AT   (451) AAGTGATCCCGCTCGTAGCCGTGCAAGTAACCGTTTTTCCTAACCGTGGC
   Gla5AT     (1) --------CCGCTCGTAGCCGTGCAAGTAACCGTTTTTCCTAACCATGGC
Consensus   (451)         CCGCTCGTAGCCGTGCAAGTAACCGTTTTTCCTAACC TGGC
                  501                                            550
    Gt5AT   (501) ATAGCCGTGGCTCTGACGGCACATCATTCAATTGCAGATGCTAAAGTTT
   Gla5AT    (43) ATAGCCGTGGCTCTGACGACGCATCACTCAATTGCAGATGGAAGAAGTGC
Consensus   (501) ATAGCCGTGGCTCTGACG C CATCA TCAATTGCAGATG  A AAGT
                  551                                            600
    Gt5AT   (551) TGTAATGTTCATCAATGCTTGGGCCTATATTAACAAATTTGGGAAAGACG
   Gla5AT    (93) TGTAAAGTTCATGAATGCTTGGGCCTATATTAACAAATTTGGAAAAGAAG
Consensus   (551) TGTAA GTTCAT AATGCTTGGGCCTATATTAACAAATTTGG AAAGA G
                  601                                            650
    Gt5AT   (601) CGGACTTGTTGTCCGCGAATCTTCTTCCATCTTTCGATAGATCGATAATC
   Gla5AT   (143) CAGACTTATTGTGTAGAAATCTTCTTCCATCTTTTGATAGATCCATAATC
Consensus   (601) C GACTT TTGT     AATCTTCTTCCATCTTT GATAGATC ATAATC
                  651                                            700
    Gt5AT   (651) AAAGATCTGTATGGCCTAGAGGAAACATTTTGGAACGAAATGCAAGATGT
   Gla5AT   (193) AAAGATCCGTATGGCCTAGAGGAAATATTTTGGAACGAAATGCAAGATAT
Consensus   (651) AAAGATC GTATGGCCTAGAGGAAA ATTTTGGAACGAAATGCAAGAT T
                  701                                            750
    Gt5AT   (701) TCTTGAAATGTTCTCTAGATTTGGAAGCAAACCCCCTCGATTCAACAAGG
   Gla5AT   (243) TCTCGAATTGTTCTCTAGATTTGGAACCAAACCCCCTCGATTCAACAAGG
Consensus   (701) TCT GAA TGTTCTCTAGATTTGGAA CAAACCCCCTCGATTCAACAAGG
                  751                                            800
    Gt5AT   (751) TACGAGCTACATATGTCCTCTCCCTTGCTGAAATCCAGAAGCTAAAGAAC
   Gla5AT   (293) TACGAGCCACATATGTACTATCCCTTGTTGAAATCGAGAGGCTAAAGAAC
Consensus   (751) TACGAGC ACATATGT CT TCCCTTG TGAAATC AGA GCTAAAGAAC
                  801                                            850
    Gt5AT   (801) AAAGTACTGAATCTCAGAGGATCCGAACCGACAATACGTGTAACGACGTT
   Gla5AT   (343) AAAGTACTGAATCTCAGAGGATGTGAACCAACAATACGCGTAACAACATT
Consensus   (801) AAAGTACTGAATCTCAGAGGAT  GAACC ACAATACG GTAAC AC TT
                  851                                            900
    Gt5AT   (851) CACAATGACGTGTGGATACGTATGGACATGCATGGTCAAATCAAAAGATG
   Gla5AT   (393) CACAGTGACATGTGGATACATATGGACATGCATGGTTAAATCAAAAGAAG
Consensus   (851) CACA TGAC TGTGGATAC TATGGACATGCATGGT AAATCAAAAGA G
                  901                                            950
    Gt5AT   (901) ACGTCGTATCAGAGGAATCATCGAACGACGAAAATGAGCTCGAGTACTTC
   Gla5AT   (443) GCAT---ATCAGAGGAATCATCAAACGACAAAAATGAGCTCGAGTACTTC
Consensus   (901)  C T   ATCAGAGGAATCATC AACGAC AAAATGAGCTCGAGTACTTC
                  951                                           1000
    Gt5AT   (951) AGTTTTACAGCGGATTGCCGAGGACTTCTGACGCCCCCGTGTCCGCCTAA
   Gla5AT   (490) AGTTTTACAGCTGATTGCCGAGGGCTTTTGACGCCCCGGTGTCCACCTAA
Consensus   (951) AGTTTTACAGC GATTGCCGAGG CTT TGACGCCCC GTGTCC CCTAA
```

```
                    1001                                              1050
    Gt5AT   (1001)  CTACTTTGGCAACTGTCTTGCGTCATGCGTTGCAAAAGCAACACATAAAG
   Gla5AT    (540)  CTACTTTGGAAACTGTCTTGCGCCGTGCCTTGCAAAAGCAACACATAAAG
Consensus   (1001)  CTACTTTGG AACTGTCTTGCG C TGC TTGCAAAAGCAACACATAAAG
                    1051                                              1100
    Gt5AT   (1051)  AGTTAGTTGGGGATAAAGGGCTTCTTGTTGCAGTTGCAGCTATTGGAGAA
   Gla5AT    (590)  AGTTAATCGGAAATAAAGGGTTTCTTGTTGCAGTTGCAGCTGTTGGGGAT
Consensus   (1051)  AGTTA T GG  ATAAAGGG TTCTTGTTGCAGTTGCAGCT TTGG GA
                    1101                                              1150
    Gt5AT   (1101)  GCCATTGAAAAGAGGTTGCACAACGAAAAAGGCGTTCTTGCAGATGCAAA
   Gla5AT    (640)  GCCATTGAAAAGAGGGTGCAGAACAAAGAAGGTGTTCTTGCAGATGCAAA
Consensus   (1101)  GCCATTGAAAAGAGG TGCA AAC AA AAGG GTTCTTGCAGATGCAAA
                    1151                                              1200
    Gt5AT   (1151)  AACTTGGTTATCGGAATCTAATGGAATCCCTTCAAAAAGATTTCTCGGGA
   Gla5AT    (690)  AACTTGGTTATCAGAATCTAAAGGAATCCCTTCAGAAAGATTGCTCGGGA
Consensus   (1151)  AACTTGGTTATC GAATCTAA GGAATCCCTTCA AAAGATT CTCGGGA
                    1201                                              1250
    Gt5AT   (1201)  TTACCGGATCGCCTAAGTTCGATTCGTATGGTGTAGATTTTGGATGGGGA
   Gla5AT    (740)  TTAGTGGATCGCCTAAGTTCGATTCGTATGGTGTAGATTTTGGATGGGGA
Consensus   (1201)  TTA  GGATCGCCTAAGTTCGATTCGTATGGTGTAGATTTTGGATGGGGA
                    1251                                              1300
    Gt5AT   (1251)  AAGCCTGCAAAATTTGACATTACCTCTGTTGATTATGCAGAATTGATTTA
   Gla5AT    (790)  AAGCCTGCAAAATTTGA---------------------------------
Consensus   (1251)  AAGCCTGCAAAATTTGA
```

S3 Fig. Alignments of partial *5GT* and *5AT* cDNA sequences encoding UDP-glucose:flavonoid 5-*O*-glucosyltransferase (5GT; A) and anthocyanin 5-aromatic acyltransferase (5AT; B) between *Gentiana triflora* (*Gt*) and *G. lutea* L. var. *aurantiaca* (*Gla*). The underlined cDNA sequences indicate the primers used to isolate cDNA fragments from petals of *G. lutea* L. var. *aurantiaca*. Gaps are inserted with a dash (-) in one of the sequences. Abbreviations: Gt, *Gentiana triflora*; Gla, *G. lutea* L. var. *aurantiaca*; 5GT, *UDP-glucose:flavonoid 5-O-glucosyltransferase gene*; 5AT, *anthocyanin 5-aromatic acyltransferase gene*. GenBank accession numbers: Gt5GT, AB363839; Gt5AT, AB010708. The partial cDNA sequences of *5GT* and *5AT* genes from *G. lutea* L. var. *aurantiaca* have been isolated by the authors in this study.
