# Supplementary material for: Differential accumulation of pelargonidin glycosides in petals at three different developmental stages of the orange-flowered gentian (Gentiana lutea L. var. aurantiaca)
Source: PLoS One. 2019 Feb 11;14(2):e0212062. doi: 10.1371/journal.pone.0212062 (PMC6370212; doi:10.1371/journal.pone.0212062)
Supplement: S4 Fig — Alignments of the deduced amino acid sequences encoded by UDP-glucose:flavonoid 5-O-glucosyltransferase (5GT) gene (A), and anthocyanin 5-aromatic acyltransferase (5AT) gene (B) from Gentiana triflora and G. lutea L. var. aurantiaca. Gaps are inserted with a dash (-) in one of the sequences. The underlined amino acid sequences from G. lutea L. var. aurantiaca were deduced from primers. Abbreviations: Gt, Gentiana triflora; Gla, G. lutea L. var. aurantiaca; 5GT, UDP-glucose:flavonoid 5-O-glucosyltransferase; 5AT, anthocyanin 5-aromatic acyltransferase. GenBank accession numbers: Gt5GT, AB363839; Gt5AT, AB010708. The partial amino acid sequences of 5GT and 5AT of G. lutea L. var. aurantiaca were deduced from partial cDNA sequences cloned by the authors in this study. (PDF) [file pone.0212062.s004.pdf]

A

```
                 101                                              150
    Gt5GT  (101) RDIIHSTKKGQGQGQGQGQGQGQGQGHPITRILYTTLLPWAADVAREFRL
   Gla5GT    (1) ------------------------------------LLPWAADVAREFRI
Consensus  (101)                                     LLPWAADVAREFRI
                 151                                              200
    Gt5GT  (151) PSVLLWTQPVTTFLTFHYYFTGYEDAINKVRNQQGTEDDSTIQLPRLPLL
   Gla5GT   (15) PSVLLWTQPVTTLVTFHYYFSGYEDAIKEVCNGT----DSTIQLPRLPLL
Consensus  (151) PSVLLWTQPVTT LTFHYYFSGYEDAI  V N      DSTIQLPRLPLL
                 201                                              250
    Gt5GT  (201) SSRDLHSFMLPSNPFKGAINTFKEHLEALDAEETPPTILVNSYDALEEEA
   Gla5GT   (61) SSRDLHSFMLPSNPFKGALKTFKEHLEALDAEQNP-TIIVNSYDALEEEA
Consensus  (201) SSRDLHSFMLPSNPFKGAI TFKEHLEALDAE  P TIIVNSYDALEEEA
                 251                                              300
    Gt5GT  (251) LQAMIPKYKTMGIGPLIPSSVFDTRETTCEVVSLVPDLAQKSKDDCQWHG
   Gla5GT  (110) LQA-IPKYKTMGIGPLIPSSIFDTKETS--EVSLVAYLLQKSKD---CHG
Consensus  (251) LQA IPKYKTMGIGPLIPSSIFDTKETS   VSLV  L QKSKD    HG

                 301                                              350
    Gt5GT  (301) WLNSKAEGSVIYVSFGSHVKQSKAQTEEIAKGLLASGHPFLWVITSNEEE
   Gla5GT  (154) WLNSKPQGSVIYVSFGSHVKQSKSQTEEIAKGLLASGHPFLWVITSNEEE
Consensus  (301) WLNSK  GSVIYVSFGSHVKQSKAQTEEIAKGLLASGHPFLWVITSNEEE
                 351                                              400
    Gt5GT  (351) --EGDEIMEQNLVEEIQEKGMMIVPWCAQFQVLKHPSVGCFMTHCGWNST
   Gla5GT  (204) AEKGKVLMERNLLKEIQEKGM-IVPWCAQIQVLKHHSVGCFMTHCGWNST
Consensus  (351)    G  IME NLL EIQEKGM IVPWCAQ QVLKH SVGCFMTHCGWNST
                 401                                              450
    Gt5GT  (399) LESIACGVPMIGFPKMFDQPTISKLIAHVWKVGVRVNAAVDGIVGQEVIK
   Gla5GT  (253) LES-----------------------------------------------
Consensus  (401) LES
```

B

```
                 151                                              200
    Gt5AT  (151) IPLVAVQVTVFPNRGIAVALTAHHSIADAKSFVMFINAWAYINKFGKDAD
   Gla5AT    (1) -PLVAVQVTVFPNHGIAVALTTHHSIADGRSAVKFMNAWAYINKFGKEAD
Consensus  (151)  PLVAVQVTVFPN GIAVALT HHSIADAKS V FINAWAYINKFGKDAD
                 201                                              250
    Gt5AT  (201) LLSANLLPSFDRSIIKDLYGLEETFWNEMQDVLEMFSRFGSKPPRFNKVR
   Gla5AT   (50) LLCRNLLPSFDRSIIKDPYGLEEIFWNEMQDILELFSRFGTKPPRFNKVR
Consensus  (201) LL  NLLPSFDRSIIKD YGLEE FWNEMQDILELFSRFGSKPPRFNKVR
                 251                                              300
    Gt5AT  (251) ATYVLSLAEIQKLKNKVLNLRGSEPTIRVTTFTMTCGYVWTCMVKSKDDV
   Gla5AT  (100) ATYVLSLVEIERLKNKVLNLRGCEPTIRVTTFTVTCGYIWTCMVKSKEGI
Consensus  (251) ATYVLSL EI KLKNKVLNLRG EPTIRVTTFTMTCGYIWTCMVKSKD I
                 301                                              350
    Gt5AT  (301) VSEESSNDENELEYFSFTADCRGLLTPPCPPNYFGNCLASCVAKATHKEL
   Gla5AT  (150) -SEESSNDKNELEYFSFTADCRGLLTPRCPPNYFGNCLAPCLAKATHKEL
Consensus  (301)  SEESSND NELEYFSFTADCRGLLTP CPPNYFGNCLA CLAKATHKEL
                 351                                              400
    Gt5AT  (351) VGDKGLLVAVAAIGEAIEKRLHNEKGVLADAKTWLSESNGIPSKRFLGIT
   Gla5AT  (199) IGNKGFLVAVAAVGDAIEKRVQNKEGVLADAKTWLSESKGIPSERLLGIS
Consensus  (351) IG KG LVAVAAIGDAIEKRL N  GVLADAKTWLSES GIPS R LGIS
                 401                                              450
    Gt5AT  (401) GSPKFDSYGVDFGWGKPAKFDITSVDYAELIYVIQSRDFEKGVEIGVSLP
   Gla5AT  (249) GSPKFDSYGVDFGWGKPAKF------------------------------
Consensus  (401) GSPKFDSYGVDFGWGKPAKF
```

S4 Fig. Alignments of the deduced amino acid sequences encoded by UDP-glucose:flavonoid 5-*O*-glucosyltransferase (5GT) gene (A), and anthocyanin 5-aromatic acyltransferase (5AT) gene (B) from *Gentiana triflora* and *G. lutea* L. var. *aurantiaca*. Gaps are inserted with a dash (-) in one of the sequences. The underlined amino acid sequences from *G. lutea* L. var. *aurantiaca* were deduced from primers. Abbreviations: Gt, *Gentiana triflora*; Gla, *G. lutea* L. var. *aurantiaca*; 5GT, UDP-glucose:flavonoid 5-O-glucosyltransferase; 5AT, anthocyanin 5-aromatic acyltransferase. GenBank accession numbers: *Gt5GT*, AB363839; *Gt5AT*, AB010708. The partial amino acid sequences of 5GT and 5AT of *G. lutea* L. var. *aurantiaca* were deduced from partial cDNA sequences cloned by the authors in this study.
