# Supplementary material for: Differential accumulation of pelargonidin glycosides in petals at three different developmental stages of the orange-flowered gentian (Gentiana lutea L. var. aurantiaca)
Source: PLoS One. 2019 Feb 11;14(2):e0212062. doi: 10.1371/journal.pone.0212062 (PMC6370212; doi:10.1371/journal.pone.0212062)
Supplement: S1 Table — (PDF) [file pone.0212062.s005.pdf]

S1 Table. Node strength (ns) and network strength (NS) of gentian phenylpropanoid genes and metabolites, expressed as the average of all the |rs| yielded by a node and the average of the ns, respectively.

ID	Kind	Node strength (ns)
ANS	gene	0.84
3GT	gene	0.84
Pelargonidin 3-*O*-(6-*O*-caffeoyl-D-glucoside)-5-*O*-(6-*O*-malonyl-β-D-glucoside)	anthocyanin	0.84
DFR	gene	0.84
Dihydrokaempferol	precursor	0.83
Pelargonidin 3,5-*O*-diglucoside	anthocyanin	0.83
5AT	gene	0.82
Pelargonidin 3-*O*-[2-*O*-(6-(E)-feruloyl-β-D-glucopyranosyl)-6-*O*-(E)-p-coumaroyl-β-D-glucopyranoside]-5-O-(β-D-glucopyranoside)	anthocyanin	0.82
5GT	gene	0.81
Pelargonidin 3-*O*-rutinoside	anthocyanin	0.78
Pelargonidin 3-*O*-rutinoside-5-*O*-β-D-glucoside	anthocyanin	0.76
Pelargonidin 3-*O*-(6-*p*-coumaroyl)glucoside	anthocyanin	0.76
Pelargonidin 3-*O*-glucoside	anthocyanin	0.71
Pelargonidin 3-*O*-(6-*O*-malonyl-β-D-glucoside)-5-glucoside	anthocyanin	0.67
Pelargonidin 3-*O*-[2-*O*-(6-(E)-feruloyl-β-D-glucopyranosyl)-6-*O*-(E)-caffeoyl-β-D-glucopyranoside]-5-*O*-(β-D-glucopyranoside)	anthocyanin	0.66
Pelargonidin 3-*O*-(6-*O*-malonyl-β-D-glucoside)	anthocyanin	0.61
Pelargonidin 3-*O*-(6-*p*-coumaroyl-D-glucoside)-5-(4-*O*-malonylglucoside)	anthocyanin	0.45
	**Network strength (NS)**	0.76
